# Manageable Bubble Release Through 3D Printed Microcapillary for Highly Efficient Overall Water Splitting

**DOI:** 10.1002/advs.202207495

**Published:** 2023-02-24

**Authors:** Tianbiao Zeng, Binbin Guo, Zhiyao Xu, Funian Mo, Xiaoteng Chen, Liping Wang, Yihong Ding, Jiaming Bai

**Affiliations:** ^1^ Key Laboratory of Carbon Materials of Zhejiang Province Wenzhou Key Lab of Advanced Energy Storage and Conversion Zhejiang Province Key Lab of Leather Engineering College of Chemistry and Materials Engineering Wenzhou University Wenzhou Zhejiang 325035 P. R. China; ^2^ Shenzhen Key Laboratory for Additive Manufacturing of High‐performance Materials Department of Mechanical and Energy Engineering Southern University of Science and Technology Shenzhen 518055 P. R. China; ^3^ Shenzhen Key Laboratory of Flexible Printed Electronics Technology Center Harbin Institute of Technology Shenzhen 518055 P. R. China

**Keywords:** 3D printing, bubbles release management, capillary force, water splitting

## Abstract

Porous metal foams (e.g., Ni/Cu/Ti) are applied as catalyst supports extensively for water splitting due to their large specific area and excellent conductivity, however, intrinsic bubble congestion is unavoidable because of the irregular three‐dimensional (3D) networks, resulting in high polarization and degraded electrocatalytic performances. To boost the H_2_O decomposition kinetics, the immediate bubble removal and water supply sequential in the gas–liquid–solid interface is essential. Inspired by the high efficiency of water/nutrient transport in the capillaries plants, this work designs a graphene‐based capillary array with side holes as catalyst support to manage the bubble release and water supply via a *Z*‐axis controllable digital light processing (DLP) 3D printing technology. Like planting rice, a low‐cost, high‐active CoNi carbonate hydroxide (CoNiCH) is planted on support. A homemade cell can reach 10 mA cm^−2^ in 1.51 V, and be kept at 30 mA cm^−2^ for 60 h without noticeable degradation, surpassing most of the known cells. This research provides a promising avenue to design and prepare advanced catalysts in various fields, including energy applications, pollutant treatment, and chemical synthesis.

## Graphical abstract

Inspired by the high efficiency of water/nutrient transport in the capillaries plants, a graphene‐based capillary array with side holes as catalyst support is designed to manage the bubble release and water supply via a *Z*‐axis controllable digital light processing (DLP) three‐dimensional (3D) printing technology, which overcomes the contradiction of bubble release/H_2_O flow in current 3D catalytic electrodes.

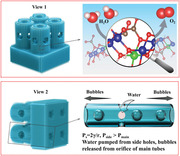

## 1 Introduction

With the continuous deterioration of the environment and increases in energy needs, hydrogen energy was recognized as one of the cleanest and most renewable substitutes for fossil fuels.^[^ ^1^, ^2^ ^]^ Electrolytic water is one of the most feasible ways to produce hydrogen, however, it only accounting ≈4% of global hydrogen production.^[^ ^3^ ^]^ Hydrogen evolution reaction (HER) and oxygen evolution reaction (OER) are two half‐cell reactions, and OER is the dynamic limit reaction.^[^ ^4^ ^]^ To accelerate the reaction process of OER, the utilization of catalysts is indispensable. Noble metals based catalysts (i.e., IrO_2_, RuO_2_, Pt, etc.) exhibit excellent catalytic activity in OER, however, their scarce natural resources and high costs block the applications.^[^ ^5^, ^6^, ^7^ ^]^ Noble metal free based catalysts, i.e., transition metals and their compounds/composites, fell into the research scope due to natural abundance and low toxicity.^[^ ^8^, ^9^ ^]^ A lot of work has been devoted to developing high‐active catalytics by regulating the components/proportions, modifying the synthesis process, and enhancing the exposed active sites.^[^ ^10^, ^11^, ^12^ ^]^ For a given catalyst, the more exposure of the active sites, the better the catalyst performance, thereby accelerating productivity. To get large exposure of the active sites, dispersing the catalyst to a layer with high mass loading is usually needed, therefore, three‐dimensional (3D) catalyst supports fall into the research scope.^[^ ^13^, ^14^ ^]^ It should be noticed that bubble congestion and electrode polarization will reduce the efficiency of the catalyst under high current density due to the insulation of bubbles. Several strategies were adopted to accelerate the bubble removal, including surface engineering and fabricating the catalyst support with 3D regular bubble transportation channels. For example, Márquez et al. regulate the surface properties of nylon by electrochemical deposition of Cu/Ni/NiFe, the overpotential of Nylon/Cu/Ni/NiFe 3D lattice was only ≈300 mV at 10 mA cm^−3^ with 85% IR compensation, but the Ni oxidation in KOH solution is unavoidable.^[^ ^15^ ^]^ The buoyancy force was also used to accelerate the bubble removal, i.e., Kou et al. designed a periodic porous of carbon doped NiO nanorods coated 3D Ni foam electrodes, showing low overpotential than carbon doped NiO nanorods coated Ni foams.^[^ ^16^ ^]^ Zhou et al. fabricated NiFe‐P_zn_ decorated Ni‐tubes sponge array, the overpotential of OER in 1 mol L^−1^ KOH was only 172 mV with 85% IR compensation.^[^ ^17^ ^]^ To utilize the buoyancy force, the electrodes are needed to orient the nozzle upwards. Besides, the flowing liquid was also applied to accelerate the bubble removal, i.e., Yang et al. designed a flow cell by using Ni/Cu nanowire felt as the electrode, exhibiting a high current density of 25 A cm^−2^ over 100 h without noticeable degradation.^[^ ^18^ ^]^ However, a drive pump is needed to maintain water flow, but the water is usually in a static state in practical application. We noticed that H_2_O/nutrient can efficiently be transported in the plant due to the capillary force, believed that capillary force can be applied to manage the bubble release and H_2_O supply in electrolytic water, thereby boosting the activity of catalysts. Unfortunately, 3D supports with plant‐like capillary geometries cannot be controllably fabricated by conventional methods. 3D printing is an effective way to design geometry in a layer‐by‐layer manner, exhibits various advantages in high geometry freedom and handle ability on construction architectures, and was regarded as an emerging material manufacturing technology. For example, Peng et al.^[^ ^19^ ^]^ printed a 24‐layer graphene/carbon nanotubes (CNTs) skeleton for catalytic support, and grew NiFeP sheets for overall water splitting, as a result, the as‐prepared electrode showed a low overpotential of 214 and 133 mV in OER and HER to reach 30 mA cm^−2^. However, extrusion‐based 3D printing technologies show several limitations in terms of unable structural precisely design on the *Z*‐axis and low dimension resolution, which was unsuitable for printing the catalyst supports with capillary geometries.

In recent years, the digital light processing (DLP) 3D printing technique has been utilized to manufacture polymeric‐based architectures with hyperfine structures that benefited from the high dimension resolution and production rate, in which a UV light with a specific wavelength was employed to initiate a photopolymerization reaction in DLP 3D process.^[^ ^20^, ^21^ ^]^ Unfortunately, the low electronic conductivity of the polymeric‐based texture blocks the application of resin‐based products in electronic‐based fields, despite the conductivity can be enhanced by heat treatment. Furthermore, the low mechanical strength of heat‐treated polymeric‐based architectures is another factor that hinders the application. Graphene is an excellent electronic conductor, however, high UV power and long exposure time are needed for DLP 3D printing due to the super light‐absorption property, which is a disadvantage to DLP 3D processes. One promising strategy for improving the electrical conductivity and mechanical strength is the introduction of graphene oxide followed by a feasible thermal reduction process,^[^ ^22^, ^23^, ^24^ ^]^ however, commercial graphene oxide showed poor dispersion ability in an organic solvent in DLP 3D printing processes, requiring functionalization of the graphene oxide (fGO) to enhance the operability.

In this work, graphene‐based catalytic support of capillary arrays with side holes was fabricated through DLP 3D printing. Bimetallic hydroxides/carbonate hydroxides are proven to be the high‐efficient catalytic for overall water splitting, and Co/Ni‐based hydroxides/carbonate hydroxides have been broadly investigated in water splitting due to easy synthesizing. Nai et al. proved that Co(OH)_2_ is better than Ni(OH)_2_ in OER, and the NiCo_2.7_(OH)*_x_* surpassed the Co(OH)_2_ and Ni(OH)_2_, illustrating the small amount of Ni substitute Co site can improve the catalytic performances dramatically.^[^ ^25^ ^]^ Kang et al. improved the catalyst performance by using the Co carbonate hydroxide instead of the hydroxide and decreased the overpotential by using a small amount of Ni substituted Co.^[^ ^26^ ^]^ Herein, like planting rice, CoNiCH was planted on support and used as the catalytic electrode for OER/HER, as a result, the as‐prepared electrode showed one of the best OER/HER performances, i.e., the lowest overpotential or highest gas production rate than the state‐of‐the‐art catalysts. Fixed the current density on 30 mA cm^−2^, the overpotential was only 207 mV for OER and 150 mV for HER. In overall H_2_O splitting, the gas release ratio reached 9.667 × 10^−4^, 1.496 × 10^−3^, and 2.204 × 10^−3^ mL cm^−2^ s^−1^ in 10, 20, and 30 mA cm^−2^, surpassing most of the advanced catalysts. For the first time, the management of bubble release/H_2_O supply by capillary force was revealed via an in situ optical microscope, in which O_2_/H_2_ was released from the orifice of main tubes, where H_2_O was flowed into capillary from the side holes. This work exampling of improving catalyst performance by introducing capillary force, and the results may trigger the application of capillary force in design/synthesis materials with advanced functions.

## 2 Results and Discussion

The H_2_O/nutrient transport on plants was observed via a water transportation experiment (Figure S1, Supporting Information), rhodamine molecule was transported to every corner of a willow branch within 10 h. Scanning electron microscope (SEM) images revealed that a large number of capillaries were existed in the willow's branches (Figure S2, Supporting Information). A water rising experiment in a capillary tube proved the capillary is the H_2_O/nutrient transport channel of plants (Figure S3, Supporting Information). Guided by the plant bionics, 3D capillaries with side holes for catalyst support were designed, in which the capillary force was introduced to manage the bubble release/H_2_O supply. Owing to the super electrical conductivity, high Young's modulus, and lightweight, graphene has been widely applied in energy‐related fields (Figure S4, Supporting Information). However, it is difficult to disperse graphene in light curable resin due to the strong Van der Waals interactions between graphene nanosheets. Additionally, owing to the black color, graphene‐based slurries require large exposure intensity to UV cure, significantly expanding the operating time. As an alternative, graphene oxide was introduced to increase the dispersing compatibility in the photocurable resins because of the oxygen‐containing groups. Note that obvious sedimentation was observed in the 5 wt% graphite oxide slurry (Figure S5a, Supporting Information), which was ascribed to the limited dispersion of graphite oxide with a high concentration.^[^ ^24^, ^27^ ^]^ After the graphite oxide was functionalized, functionalized graphene oxide (fGO) suspensions with various concentrations exhibited superior dispersion stability for 24 h (Figure S5b, Supporting Information). FT‐IR shown in Figure S6 (Supporting Information) illustrates BYK was adsorbed on graphite oxide after functionalization, and the TGA results manifested that BYK accounted for 11.3 wt% in fGO (Figure S7, Supporting Information). Compared to graphite oxide, the thickness of fGO was decreased to 1/10 of graphite oxide (Figures S8–S9, Supporting Information). Besides, the rheology of fGO suspension was investigated, found that 10 wt% fGO contained ink exhibited a moderate viscosity, which was suitable for DLP 3D printing (Figure S10, Supporting Information).

**Figure 1:**
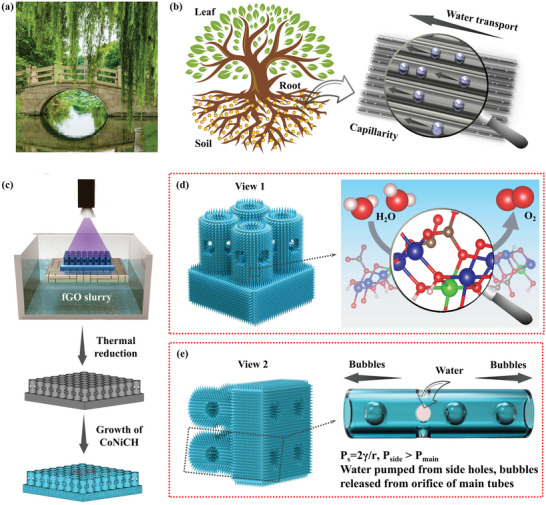
a) Digital camera photo of a willow growing near the water (Photo credit: Unsplash/CC0 Public Domain), b) illustrate H_2_O transport in a plant, c) schematic of the fabrication of 5# and 5#N, d) aeroview of 5#N, and schematic the H_2_O splitting in the catalytic of CoNiCH, e) side oblique view of 5#, and illustrate the mechanism of bubble release from the orifice of main tube/H_2_O supplied from side holes.

**Figure** **1**a shows a digital camera photo of a willow growing near the water, and Figure 1b illustrates the H_2_O transported in the willows’ inner capillaries. Based on this magical phenomenon, the capillaries array with side holes as the catalyst supports were designed and fabricated, and growing the low‐cost CoNiCH catalysts for the OER/HER electrodes, as illustrated in Figure 1c. The inner channels of the capillaries penetrated the substrate, and all the surface were covered by the low‐cost, high‐active CoNiCH nanoneedles catalysts (Figure 1d,e). For comparison, several catalyst supports including a solid slab, slab with holes array, column array, and tubes array without side holes were designed and grew the CoNiCH nanoneedles on surfaces (Figure S11a, Supporting Information). To simplify the narration, those supports were denoted to *n*# (*n* = 1–5), where *n* = 1, 2, 3, 4, and 5 represent the solid slab, slab with holes, column array, tubes array without side holes, and tubes array with side holes, and the parameters of *n*# are listed in Table S1 (Supporting Information), the supports grown with CoNiCH nanoneedles were denoted to *n*#N, respectively, where *N* represents the CoNiCH nanoneedles.

Fixed the length and width to 8.0 mm, the surface area of *n*# was 137.6, 182.9, 307.5, 466.1, and 450.4 mm^2^ for *n* = 1, 2, 3, 4, and 5, respectively, with the corresponding volume of 19.2, 15.8, 31.9, 22.9, and 21.5 mm^3^. The surface‐to‐area and porosity are plotted and shown in Figure S11b (Supporting Information), it can see that 5# reached the highest surface‐to‐area and porosity.

To investigate the morphology of various catalyst supports, SEM images were collected. As shown in Figure S12a,b, (Supporting Information), 1# was a flat plate with a large number of irregular micro holes on the surface, and those holes originated from the pyrolysis of the fGO/resin component, which created a rough surface of the support and very advantage to anchoring the CoNiCH. In #2, neatly arranged designed mesoscopic holes array were observed (Figure S12c,d, Supporting Information). In the situation of #3, #4, and #5, neatly arranged designed mesoscopic columns array, tubes array without side holes, and tubes array with side holes were observed (Figure S12e–l, Supporting Information). The side holes with a diameter of 30 µm in capillaries’ of 5# can be observed clearly, and also detected via a reconstructed 3D computed tomography (3D CT, Figure S13, Supporting Information), proving the successful fabrication of the target catalyst support. The diameter of the holes in 2# is 80 µm, and the distance of the two adjacent holes’ centers was 220 µm (Figure S14a, Supporting Information). For #3, the diameter of columns and distances of two adjacent columns’ centers were 180 and 250 µm, respectively (Figure S14b, Supporting Information). In the situation of #4 and #5, the outer diameter, thickness, and distances of the two adjacent tubes center were 160, 40, and 220 µm, respectively (Figure S14c,d, Supporting Information). The structural parameters were about 38% shrinkage compared to without heat treatment of newly printed 3D structures (Figure S15, Supporting Information). A nanoindentation test was performed to investigate the hardness of those catalyst supports, as expected, the hardness exceeded 31.75±8.34 MPa, and the indentation modulus exceeded 55.35±5.3 MPa (Figure S16, Supporting Information), which was strong enough to withstand the bubble impingement during OER/HER processes.

**Figure 2:**
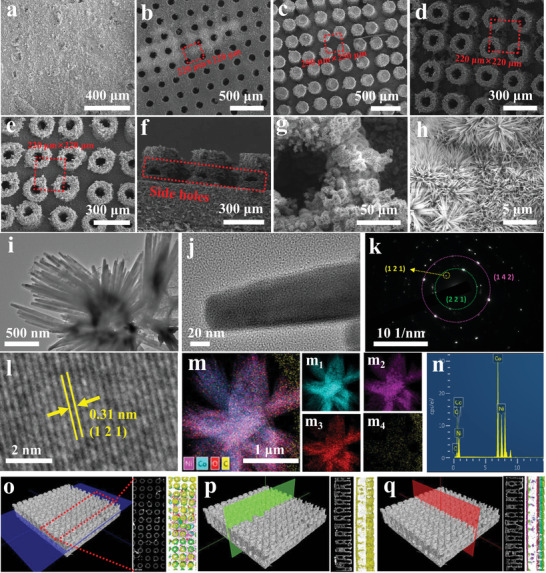
Scanning electron microscope (SEM) images of 1#N (a), 2#N (b), 3#N (c), 4#N (d), 5#N (e–h), TEM images of CoNiCH (i,j,l), selected area electron diffraction (SAED) image of CoNiCH (k), Co, Ni, C, O elements distribution images of CoNiCH (m), EDS image of CoNiCH (n), three‐dimensional (3D) computed tomography (CT) images of 5#N in the top (o), front (p) and left view (q).

Like planting rice, CoNiCH was planted on *n*# successfully, and structural parameters of *n*#N were kept constant with *n*# (**Figure** **2**a–e). Notice that side holes with a diameter of 30 µm in 5#N still can be observed, despite some CoNiCH grown on the surface of the inner wall (Figure 2f,g). The length of CoNiCH reached ≈5 µm estimated from a high‐resolution SEM image (Figure 2h), with a diameter of 30–70 nm distinguished from TEM images (Figure 2i,j). The electron diffraction spots of (1 2 1), (2 2 1), and (1 4 2) planes generated from CoNiCH can be distinguished from the selected area electron diffraction (SAED) image (Figure 2k), and crystal lattice of (1 2 1) was observed in the high‐resolution TEM image (Figure 2l). Elements mapping images and energy dispersive spectroscopy shown in Figure 2m,n illustrate the Co, Ni, O and C existence in CoNiCH and uniform distribution. XRD and XPS analysis shown in Figure S17 (Supporting Information) proved that Ni has substituted the Co atoms of Co(CO_3_)_0.5_(OH) ·0.11H_2_O, instead of forming the composite.^[^ ^28^, ^29^, ^30^ ^]^ A detailed investigation by SEM proves all the supports’ surface was planted CoNiCH successfully (Figure S18, Supporting Information). The CoNiCH loaded on *n*#N was in the range of 3.7–4.7 mg cm^−2^ (Table S2, Supporting Information). Catalyst distribution can also be acquired via a reconstructed 3D CT. Figure 2o–q shows the top, front, and left view of 5#N, with yellow color rendering the catalyst support, and green color rendering the CoNiCH catalyst. The attachment of green on yellow proves the support was covered with CoNiCH catalysts.

**Figure 3:**
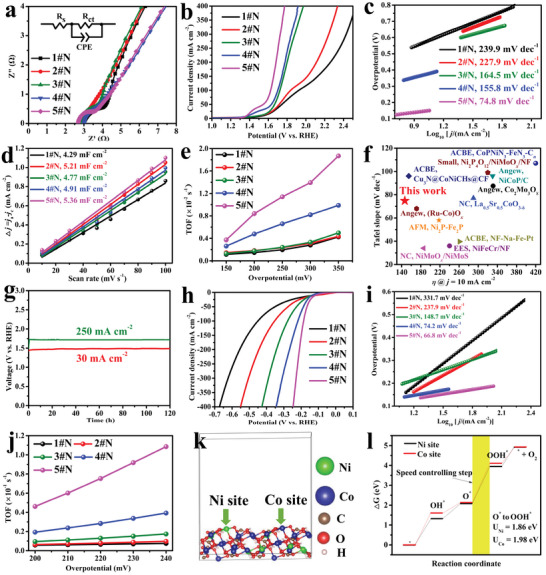
a–g) Oxygen evolution reaction (OER) performances of *n*#N. a) Electrochemical impedance spectroscopy (EIS) curve with an inserted fitting circuit diagram, b) linear sweep voltammetry (LSV), c) Tafel plots, d) double‐layer capacitance analysis, e) turnover frequency (TOF), f) comparison of the overpotential and Tafel slope of 5#N at 10 mA cm^−2^ with state‐of‐the‐art electrocatalysts, g) *i*–*t* curve test in 30 and 250 mA cm^−2^. h–j) Hydrogen evolution reaction (HER) performances. h) LSV, i) Tafel plots, j) TOF. k) (1 2 1) crystal lattice plane of CoNiCH supercell, l) Gibbs free energy of OER in Ni and Co site. The abbreviation in figure (f) is as follows: NC: Nature communications, EES: Energy and Environmental Science, ACBE: Applied Catalysis B: Environmental, AFM: Advanced Functional Materials, Angew: Angewandte Chemie International Edition.

OER is the rate‐determining step of electrolytic water, herein, *n*#N was investigated in a 1.0 mol L^−1^ NaOH aqueous solution using a three‐electrode system. The reference voltage was corrected to reversible hydrogen electrode (RHE) potential, and the test was operated in a static electrolyte, that without any stirring. **Figure** **3**a shows the electrochemical impedance spectroscopy (EIS) curves, the solution impedance (*R*_s_) was less than 3 Ω, indicating the high conductivity of those *n*#N electrodes. Linear sweep voltammetry (LSV) curves scanned at 10 mV s^−1^ with a 90% IR compensation are shown in Figure 3b. 5#N required only 147, 207, and 322 mV to achieve 10, 30 and 50 mV cm^−2^, respectively. Fixed the current density at 50 mV cm^−2^, the overpotential of 1#N, 2#N, 3#N, and 4#N were 528, 501, 446, and 409 mV, respectively. Furthermore, 5#N reached 100, 150, 200, 250, 300, and 350 mA cm^−2^ in the overpotential of 395, 427, 453, 475, 498, and 523 mV, respectively. The Tafel slope of 5#N was 74.8 mV dec^−1^ (Figure 3c), lower than that of the 1#N (239.9 mV dec^−1^), 2#N (227.9 mV dec^−1^), 3#N (164.5 mV dec^−1^), and 4#N (155.8 mV dec^−1^). Notice that the CV scan current density of 5#N in 10 mV s^−1^ is only 0.205 mA cm^−2^ at 1.23 V versus RHE, illustrating the current density of the LSV curve contributed by the capacitance was about 0.205 mA cm^−2^. Even at the low current density of around 10 mA cm^−2^, the voltage fluctuation caused by the capacitance contribution was less than 0.2 mV (i.e., at 9.778 mA cm^−2^ and 10.222 mA cm^−2^, the voltage was 1.3705 and 1.3723 V versus RHE. The variation of the current density reached ≈0.4 mA cm^−2^, but the voltage difference is less than 0.2 mV), which is negligible, thus the electrocatalytic active of the LSV didn't overrate. The double‐layer capacitance (*C*_dl_) of *n*#N calculated from rate CVs were 4.29, 5.21, 4.77, 5.91, and 5.36 mF cm^−2^ (Figure 3d, Figure S19, Supporting Information), respectively, 5#N shows the maximum active sites than other *n*#N. The intrinsic catalytic activity of the OER can be evaluated by the turnover frequency (TOF).^[^ ^17^ ^]^ Herein, the TOF at the overpotential of 150, 200, 250, 300, and 350 mV was calculated and shown in Figure 3e, 5#N exhibits the highest TOF than other *n*#N, illustrates the higher H_2_O decomposition efficiency of 5#N. We note that the catalyst activity of *n*#N abides by the following rule: 5#N>4#N>3#N>2#N>1#N. The *n*#N and *n*# were also tested the OER without IR compensation and compared it with using Ni foam as the catalyst support, considering that Ni foam is catalyst support widely used in various fields. CoNiCH decorated Ni foam (CoNiCH/Ni, Figure S21 (Supporting Information), the SEM of CoNiCH/Ni shown in Figure S20 (Supporting Information) shows a Ni oxidation peak at 1.4–1.6 V, implies the Ni foam is unstable for OER (Figure S21a,b, Supporting Information).^[^ ^15^ ^]^ Furthermore, 5#N showed lower overpotential than CoNiCH/Ni above 1.6 V (Figure S21c, Supporting Information). The Tafel slope of 5#N and CoNiCH/Ni without IR compensation was calculated and shown in Figure S21d (Supporting Information), and the Tafel slope value of 5#N is lower than that of CoNiCH/Ni. The Tafel slope value and overpotential at 10 mA cm^−2^ are usually used to compare the active of catalysis, herein, 5#N was compared with state‐of‐the‐art catalysis,^[^ ^31^, ^32^, ^33^, ^34^, ^35^, ^36^, ^37^, ^38^, ^39^, ^40^ ^]^ and as a result, 5#N show one of the best performances, i.e., lowest overpotential or Tafel slope (Figure 3f). Fixed the current density to 30 and 250 mA cm^−2^, 5#N showed negligible voltage variation in the long‐term *i*–*t* test even reach to 120 h (Figure 3g). The CV test performed on three‐electrode system in the voltage range of 0–0.9 V versus RHE also illustrated the superior electrochemical stability of 5#N (Figure S22, Supporting Information).

Next, HER was tested, and LSV with a 90% IR compensation is shown in Figure 3h. The catalytic activity of *n*#N still followed the same rules as OER. The overpotential of 100 mA cm^−2^ for *n* = 1, 2, 3, 4, and 5 were 451, 360, 338, 218, and 186 mV, respectively, with the corresponding Tafel slope of 331.7, 237.9, 148.7, 74.2, and 66.8 mV dec^−1^ (Figure 3i). Without IR compensation, the overpotential of *n*#N at 100 mA cm^−1^ was lower than 0.5 V (Figure S23, Supporting Information). The TOF of HER at the overpotential of 200–240 mV was calculated and shown in Figure 3j, proving that 5#N exhibits the highest TOF than other *n*#N.

The H_2_O decomposition mechanism on CoNiCH was investigated by DFT calculation. The (1 2 1) plane of CoNiCH was cleaved and used as the exposed surface according to the HRTEM image (Figure 2l). Figure 3k displays a Ni substituted Co model of cobalt carbonate hydroxide, and charge density difference analysis illustrates Ni atoms can substitute the Co by forming Ni−O bonds (Figure S24, Supporting Information). Bader charge analysis implies Co, Ni, C, and H were potential OER active sites, and O was the HER active site (Figure S25, Supporting Information). Figure 3l shows the Gibbs free energy of OER in Ni and Co sites. The form of superoxide radical was a speeding decisive step (detailed discussion is appended in Text S1, Supporting Information), and the overpotential of the Co site was slightly higher than the Ni site, proving Ni substitute Co was an advantage to improve the OER performances. However, the strong affinity of C−O and H−O resulting in C and H were not suitable for OER (Table S3, Supporting Information). The H adsorption‐free energy illustrated that the O surrounding Ni site was more suitable for HER (Figure S26 and Table S4, Supporting Information). The above analysis proved Ni substituted Co improved the catalyst active in overall water splitting.

**Figure 4:**
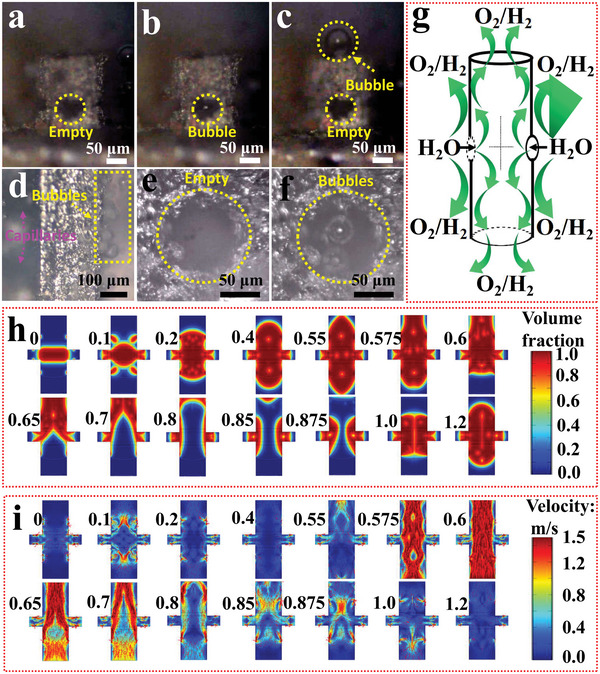
a–f) In situ optical microscope images of O_2_ release in 5#N. a–c) Side view of O_2_ released from a capillary, d) side view of O_2_ released from the back of capillaries, e,f) front view of O_2_ released from the back nozzle of a capillary. g) The H_2_O flow into capillaries from side holes, H_2_O decomposition on the outer and inner surface of the capillaries, and O_2_/H_2_ ejected from main tubes’ nozzle, h) volume fraction of H_2_O/O_2_ in a simulation period, i) the velocity of O_2_ release/H_2_O supply in a simulation period. The red region represents O_2_, the blue region is H_2_O, total simulate time was 1.2 ms.

Besides the positive role of CoNiCH on OER/HER, the advantages contributed by geometric design were also investigated via in situ optical microscope observation. Figures S27 and S28 (Supporting Information) show the digital photos of homemade cells. Both OER and HER, bubbles released from 1#N, 2#N, and 3#N tended to occur in edges, rough surfaces, and corners (Movie S1–S6, Supporting Information). In the situation of 4#N and 5#N, besides edges, rough surfaces, and corners, a large number of bubbles were released from capillaries and released faster in 5#N than in 4#N (Movie S7–S10, S12, S13, Supporting Information). In 5#N, it is worth noting that, bubbles were not released from capillaries’ side holes, but released from both sides of the main tubes’ nozzle (**Figure** **4**a–f, Figure S29, Movies S11, S14, Supporting Information). Figure 4g illustrates that H_2_O can decompose in the inner and outer surfaces of the capillaries, the H_2_O is flow into capillaries from side holes, and H_2_/O_2_ is only ejected from the main tubes’ nozzle. Based on the capillary theory, the smaller the radius curvature, the higher the additional pressure of the liquid subjected (*p*_s_ = 2*γ*/*r*).^[^ ^41^ ^]^ Herein, the *γ* of 1 moL L^−1^ NaOH was tested to 7.818 × 10^−2^ N m^−1^. Considering the radius curvature of side holes was only about 15 µm and the inner radius of the main tube was 40 µm, the net pressure of electrolyte in the nozzle of side holes reached 6.5 kPa [≈0.064 atm, 2 × 7.818 × 10^−2^ × (1/15 − 1/40) × 10^6^ Pa]. It can be deduced that H_2_O will be pushed into the capillaries from side holes, then bubbles will be pushed and released from the main tubes’ nozzle. The fast removal of bubbles avoided the congestion of insulated O_2_/H_2_ in the catalyst active sites, and the supply of H_2_O from side holes immediately ensures the catalyst is working at all times, such a working manner of separating the bubbles release and H_2_O supply channels decreased the electrode polarization, accelerated the H_2_O decomposition kinetics, and save the energy.

The benefits of capillary force on the management of H_2_O supply/bubble release, CoNiCH decorated Ni foam was used to compare the bubble release in OER/HER. In situ optical microscope observation found that the bubble with large volumes was blocked when released from the chaotic skeleton of Ni foam (Movie S15, S16, Supporting Information).

To illustrate the positive role of geometric design on OER/HER theoretically, bubble release/H_2_O supply on a capillary was simulated on Comsol. A capillary with two side holes and eight bubble occurrence points was built based on the actual size (Figure S30, Supporting Information). Figure 4h shows the O_2_ (red region) was produced and gradually ejected from the capillary orifice, and cycled around, but did not leak from side holes during the whole period. The bubble velocity and pressure reached 1.5 m s^−1^ and 2 kPa (Figure 4i and Figure S31, Supporting Information). Considering the continuous escape of the bubble from the orifice of the capillary, it can be deduced that H_2_O was pushed into the capillary from side holes continuously.

**Figure 5:**
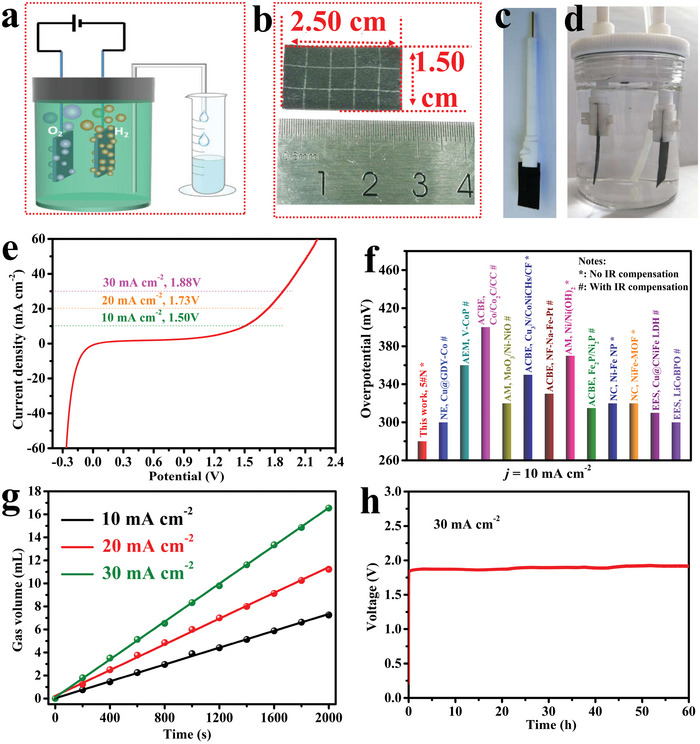
Overall water splitting evaluation of 5#N. a) Schematic of the unit cell, b) an enlarged 5#N electrode, c) a digital image of a fixed 5#N by Pt electrode clamp, d) an assembled unit cell, e) linear sweep voltammetry (LSV) curve scanned at 10 mV s^−1^ without IR compensation, f) compare the overpotential of 5#N at 10 mA cm^−2^ with state‐of‐the‐art catalytic, g) collected gas volume in a 2000 s period, h) *i*–*t* curve tested at 30 mA cm^−2^. The abbreviation in figure (f) is as follows: NE: Nano Energy, AEM: Advanced Energy Materials, ACBE: Applied Catalysis B: Environmental, AM: Advanced Materials, NC: Nature communications, EES: Energy and Environmental Science.

Based on the high HER/OER catalytic activity, the overall water splitting was evaluated to verify the potentiality of practical applications. **Figure** **5**a shows the assembly principle of a unit cell, and Figure 5b–d shows the digital image of an enlarged 5#N, a fixed 5#N, and a unit cell for overall H_2_O splitting. Only two electrodes were utilized, and the generated gas was collected by the drainage method (Figure S32, Supporting Information). LSV scanned at 10 mV s^−1^ without IR compensation is shown in Figure 5e, only 1.51, 1.72, and 1.87 V were needed to reach 10, 20, and 30 mA cm^−2^. The LSV of 3#N and 4#N were also tested (Figure S33, Supporting Information). The capillary force played a positive role in 4#N but didn't in 3#N, resulting in low overall water splitting overpotential of 4#N, and the overpotential of 5#N was decreased further due to the orderly entry of water and the escape of bubbles. The LSV curve of Pt/Pt for OER was also tested by using a plastic disc with the same thickness of 5#N as the electrode and normalized the current density with the calculation protocol of 5#N, the result is shown in Figure S34 (Supporting Information). The current density of Pt/Pt in 1.51, 1.73, and 1.87 were 0.1, 0.42, and 1.01 mA cm^−2^, respectively. In 10 mA cm^−2^, the current density contribution by Pt/Pt was negligible. The overpotential at 10 mA cm^−2^ was compared with state‐of‐the‐art catalytic (Figure 5f),^[^ ^33^, ^36^, ^42^, ^43^, ^44^, ^45^, ^46^, ^47^, ^48^, ^49^, ^50^, ^51^ ^]^ as a result, 5#N showed the lowest overpotential. In recent years, Liang et al. fabricated Ni_0.8_Fe_0.2_ oxyhydroxide@NiFe alloy nanowire array on Ni foam for water splitting, which only required 1.545 V for 100 mA cm^−2^ with 95% IR compensation, herein, the 5#N required 1.571 V for 100 mA cm^−2^ with 90% IR compensation (Figure S35, Supporting Information), the voltage difference was less than 0.03 V.^[^ ^52^ ^]^ The synthesis of Ni_0.8_Fe_0.2_ oxyhydroxide@NiFe alloy nanowire array on Ni foam needed H_2_PtCl_6_ as the nucleating agent to promote the formation of initial Ni seeds, our work provides another way to prepare high‐performance water splitting electrode without noble metal. The gas production rate of 5#N was also investigated. At a 2000 s period, 7.25, 11.22, and 16.53 mL gas (H_2_ + O_2_) were corrected under the current density of 10, 20, and 30 mA cm^−2^ (Figure 5g), respectively, and the gas release ratio reached 9.667 × 10^−4^, 1.496 × 10^−3^, and 2.204 × 10^−3^ mL cm^−2^ s^−1^, surpassing the advanced catalytic (3D printed graphene decorated with NiFeP, Cu_3_N/CoNiCH/CF, Co/Co_2_C anchored on carbon cloth, etc).^[^ ^19^, ^36^, ^44^ ^]^ The *i*–*t* test shows this unit cell driven at 30 mA cm^−2^ without any voltage attenuation even the time reached 60 h (Figure 5h). The tested electrode was further investigated by XRD, and no obvious phase deprivation in CoNiCH catalytic can be detected (Figure S36, Supporting Information). SEM images of tested 5#N prove the morphology of CoNiCH was unchangeable (Figure S37, Supporting Information). All in all, experiment results illustrate that 5#N is stable for practical application.

## 3 Conclusions

In summary, electrolytic water continues to surprise us, our results present another one. We introduce the capillary force to manage the bubble release/H_2_O supply in water splitting by designing 3D catalytic supports via *Z*‐axis controllable DLP 3D printing, through this approach, designed and fabricated solid slab, slab with holes array, columns array, tubs array without side holes and tubs array with side holes as catalytic supports, and adopted low‐cost, easy‐synthesized CoNiCH needles as the active catalytic. Bubble release/H_2_O supply orderly in 5#N was achieved and confirmed by in situ optical microscopy observation and theoretical simulation, which overcome the contradiction of bubble release/H_2_O flow in current 3D catalytic electrodes. Under the management of the capillary force, the bubble was released from the main nozzle of the capillaries, and H_2_O was supplied into the capillaries from side holes. The 5#N showed one of the best performances compared to state‐of‐the‐art catalytic, i.e., lowest overpotential and highest gas production rate. This structural design strategy overcomes the drawbacks of bubble release congestion, clarified the capillary force can be used to boost the catalyst performances of OER/HER, and it is also our hope that this catalyst design strategy may trigger the designing of advanced catalytic in various fields, including pollutant treatment, chemical synthesis, and energy applications.

## Conflict of Interest

The authors declare no conflict of interest.

## Supporting information

Supporting Information

Supplemental Movie 1

Supplemental Movie 2

Supplemental Movie 3

Supplemental Movie 4

Supplemental Movie 5

Supplemental Movie 6

Supplemental Movie 7

Supplemental Movie 8

Supplemental Movie 9

Supplemental Movie 10

Supplemental Movie 11

Supplemental Movie 12

Supplemental Movie 13

Supplemental Movie 14

Supplemental Movie 15

Supplemental Movie 16

## Data Availability

The data that support the findings of this study are available from the corresponding author upon reasonable request.
