# Supplementary material for: Manageable Bubble Release Through 3D Printed Microcapillary for Highly Efficient Overall Water Splitting
Source: Adv Sci (Weinh). 2023 Feb 24;10(13):2207495. doi: 10.1002/advs.202207495 (PMC10161030; doi:10.1002/advs.202207495)
Supplement: Supplementary file 1 — Supporting Information [file ADVS-10-2207495-s010.pdf]

ADVANCED SCIENCE
Open Access

Supporting Information

for *Adv. Sci.*, DOI 10.1002/advs.202207495

Manageable Bubble Release Through 3D Printed Microcapillary for Highly Efficient Overall Water Splitting

Tianbiao Zeng, Binbin Guo, Zhiyao Xu, Funian Mo, Xiaoteng Chen, Liping Wang, Yihong Ding and Jiaming Bai**

Supplement Information for

Manageable bubble release through 3D printed Micro Capillary for highly efficient overall water splitting

Tianbiao Zeng[a#], Binbin Guo[b#], Zhiyao Xu[b], Funian Mo[c], Xiaoteng Chen[b], Liping Wang [b], Yihong Ding[a*], Jiaming Bai[b*]

[a] Key Laboratory of Carbon Materials of Zhejiang Province, Wenzhou Key Lab of Advanced Energy Storage and Conversion, Zhejiang Province Key Lab of Leather Engineering, College of Chemistry and Materials Engineering, Wenzhou University, Wenzhou Zhejiang 325035, China

[b] Shenzhen Key Laboratory for Additive Manufacturing of High-performance Materials, Department of Mechanical and Energy Engineering, Southern University of Science and Technology, Shenzhen, 518055, China

[c] Shenzhen Key Laboratory of Flexible Printed Electronics Technology Center, Harbin Institute of Technology, Shenzhen, 518055, China

[*] Corresponding author: Yihong Ding (yhdd@wzu.edu.cn), Jiaming Bai (baijm@sustech.edu.cn)

[#] These authors contributed equally to this work.

Experimental Section

1. Materials

All purchased materials were utilized without further treatment in this work. Graphite oxide of 0.5~40 μm size scale and purity of > 97 % was purchased from Turing Evolution Technology Co., Ltd., Shenzhen, China. BYK 2152 wetting agent was supplied from BYK Chemie, Germany. Absolute ethanol was produced by Changtai Chemical Technology Co., Ltd., Shenzhen, China. Acryloylmorpholine (ACMO) and polyester acrylate were supplied by Ruiyue Trading Co., Ltd. (Guangzhou, China) and Zhongbang UV-Curable Environmental Material Co., Ltd. (Shenzhen,

China), respectively. Diphenyl (2,4,6-trimethylbenzoyl) phosphine oxide (TPO) was supplied by Macklin Biochemical Co., Ltd., Shanghai, China. NaOH, NiCl·6H_2O, CoCl·6H_2O, and $CO(NH_2)_2$ were supplied from Shanghai Aladdin Bio-Chem Technology Co., Ltd. Deionized water with 18.25 MΩ resistivity was homemade. Glass capillary of 400 and 500 μm inner diameter were supplied from Nantong Zeyuan Experimental Equipment Co., Ltd, China. Nickel foam was supplied from Hebei Ruiyun Wire Mesh Technology Co., Ltd, China.

2. Preparation of functionalized graphene oxide powders

Functionalized graphene oxide (fGO) powders were prepared by using graphite oxide and BYK as raw materials. Typically, 10.0 g graphite oxide and 80.0 g BYK were added into a beaker, then 600.0 g absolute ethanol was added and ultrasonically for 1 h. The slurry was corrected by centrifuging at 9,000 rpm for 30 min and removing the redundant BYK with absolute ethanol wash several times, then dried in the 80 °C vacuum oven.

3. Preparation of 3D catalyst supports

The 3D models of catalyst supports were drawn using SolidWorks software. 20.0 g fGO powder was mixed with 128.0 g ACMO, 50.0 g polyester acrylate, and 2.0 g TPO in a container, then ball milling in a tubular mixer with a periodic 3D motion for 12 h to prepare the 10.0 wt.% fGO slurry. A 3D printer can provide a UV laser of 405 nm wavelength, and an X/Y plane resolution of 30 μm pixels was used to print the catalyst supports. The parameters of printing were as follows: layer thickness was 10 μm, the intensity of the UV laser was 40 mW cm^{-2}, and the exposure time was 4 s. During 3D printing, radical chain-growth reactions occurred between fGO, ACMO, polyester acrylate, and TPO. After printing finished, 3D catalyst supports were washed several times with absolute ethanol to remove redundant uncured resins, then volatilization of the absolute ethanol in the atmosphere. Finally, as-printed 3D catalyst supports were heated in N_2 at 1050 °C for 3 h with a temperature-increasing slope of 2 °C min^{-1}.

4. Plant $Co_{0.75}Ni_{0.25}(CO_3)_{0.5}(OH)\cdot 0.11H_2O$ catalytic nanoneedles on 3D catalyst supports

Above heat-treated 3D catalyst supports were immersed in Co^{2+}, Ni^{2+}, $CO(NH_2)_2$ solutions to plant the $Co_{0.75}Ni_{0.25}(CO_3)$0.5(OH)·0.11H_2O catalytic nanoneedles (CoNiCH). Typically, 1.426 g

$CoCl·6H_2O$, 0.475 g $NiCl·6H_2O$, and 0.720 g $CO(NH_2)_2$ were dissolved into 40 mL H_2O, then transfer to a polytetrafluoroethylene reactor tank with a volume of 50 mL. Several 3D catalyst support discs were immersed into the solution, then the reactor tank was sealed with a stainless steel shell and heated at a 120 ℃ electric blast oven for 5 h. After heating finished and cooling down to room temperature, the discs were washed with deionized water and dried in a 60 ℃ electric blast oven to obtain target products.

5. Characterization and Electrochemical Measurements

The morphology of all samples was observed on field emission scanning electron microscopy (FESEM, Nova 200 NanoSEM, America). The surface groups of graphite oxide and fGO were studied via FTIR spectrometer (PE Spectrum 100, America), and measured the thickness via atomic force microscopy (AFM, tapping mode, Bruker’s Dimension Edge, America). Morphology, atomic arrangement, and element distribution of CoNiCH were investigated by transmission electron microscopy (TEM, JEM-2100F, Japan). The apparent viscosity of various fGO slurries was acquired on a rotational rheometer MCR-92 (Anton Paar, Australia). Nanoindentation experiments were adopted to study the structure strength of 3D support in a nanoindenter (DUH-211S, Shimadzu Co., Japan). Bubble release was in-situ observed on RuiHoge metalloscope with a 16-megapixel CCD camera (Nanjing Nanpai Technology Co., Ltd, China). The pore network of 3D support and CoNiCH distribution was studied using a high-resolution micro-computed tomography (micro-CT) system, diondo d2 (ND Inspection & Control Solution, China).

The OER, HER, and overall H_2O splitting were tested in a static electrolyte, that without any stirring. An aqueous solution of 1.0 mol L^{-1} NaOH was used as an electrolyte to evaluate the OER and HER performances. In the half-cell test, a platinum electrode with 1.0×1.5 cm^2 was used as the counter electrode, and Ag/AgCl electrode was used as the voltage reference electrode. In the overall H_2O splitting test, only two electrodes were adopted. Samples were fixed by a platinum electrode clamp. Linear sweep voltammetry (LSV) was tested on CHI 660E electrochemical workstation (Shanghai Chenhua Instrument Co., Ltd), and the voltage sweep rate was 10 mV s^{-1}. Cyclic voltammetry (CV) was scanned on EC1001B electrochemical workstation (Mesobiosystems Co., Ltd, Wuhan, China). The voltage of the working electrode of the half-cell test was calculated based on the following equation:[1]

$$E\ (RHE) = E\ (Ag/AgCl) + 0.059pH + 0.197 \qquad (V)$$

The E (RHE) is the standard hydrogen electrode potential. Herein, saturated AgCl solution was adopted in the reference electrode and 1.0 mol L^{-1} of NaOH aqueous solution was used as the electrolyte, thus,

$$E\ (RHE) = E\ (Ag/AgCl) + 1.023 \qquad (V).$$

The turnover frequency (TOF) has been calculated based on the following formula:[2]

$$TOF = \frac{j \cdot A}{4 \cdot m \cdot F}$$

where j represents the current density (A cm^{-2}) at the overpotential, A is the electrode surface area (cm^{-2}), F is the faradaic constant (96485.6 C mol^{-1}), and m is the moles of CoNiCH deposited on substrates.

6. Computational details

The first principle calculation (DFT) was performed in VASP software. Atomic arrangement models were constructed based on a previously reported $Co_2(OH)_2CO_3$ structure.[3] Generalized gradient approximation Perdew-Burke-Ernzerhof (GGA-PBE) exchange-correlation functional was employed to describe electron-orbit interaction energy. The cut-off energy was set to 500 eV, and a 4×4×1 k-point mesh was selected in the first Brillouin zone. LDA + U correction was used for Ni and Co atoms, in which U = 4.216 eV and J = 0.816 eV for Ni atoms, U = 4.216 eV, and J = 0.802 eV for Co atoms.[4, 5] The structures were read on VESTA software.

Bubble release and water flow were simulated on Comsol software based on the fluid dynamics analysis method. A capillary of 240 μm length and 80 μm inner diameter was used as the simulation model, with two smaller capillaries of 30 μm inner diameter vertically welded on both sides. Uniformly distributed 8 gas occurring points were set in the model, as shown in Fig. S34. The momentum conservation equation of H_2O/gas in the system can be described by Navier-Stokes equations:[6]

$$\rho(\mathbf{u} \cdot \nabla)\mathbf{u} = \nabla \cdot [-p\mathbf{l} + \boldsymbol{\kappa}] + \mathbf{F}$$
$$\rho\nabla \cdot \mathbf{u} = 0$$
$$\boldsymbol{\kappa} = \mu(\nabla\mathbf{u} + (\nabla\mathbf{u})^{\mathrm{T}})$$

The transport of the fluid interface separating the two phases can be described by the following

equation:

$$\frac{\partial \Phi}{\partial t} + \mathbf{u} \cdot \partial \Phi = \gamma \nabla \cdot (\varepsilon_{ls} \nabla \Phi - \Phi(1-\Phi) \frac{\nabla \Phi}{|\nabla \Phi|})$$

The outlet of H_2O/gas can be described by the following equation:

$$(\varepsilon_{ls} \nabla \Phi - \Phi(1-\Phi) \frac{\nabla \Phi}{|\nabla \Phi|}) \cdot \mathbf{n} = 0$$

Where ρ is the density of the fluid, **u** is the speed at time t, γ is the initialization parameter, in this work, set to 1 m s^{-1}, ε_{ls} is the interface thickness parameter, the default was used in this work. Based on the above equations, the surface tension of H_2O was set to 0.07 N m^{-1}, the contact angle of H_2O with a model was set to 0°, the model is divided into quadrilateral meshes of 2 μm units side length and without distortion, default parameters of H_2O, O_2 and H_2 included in the software were adopted.

Figures and Tables

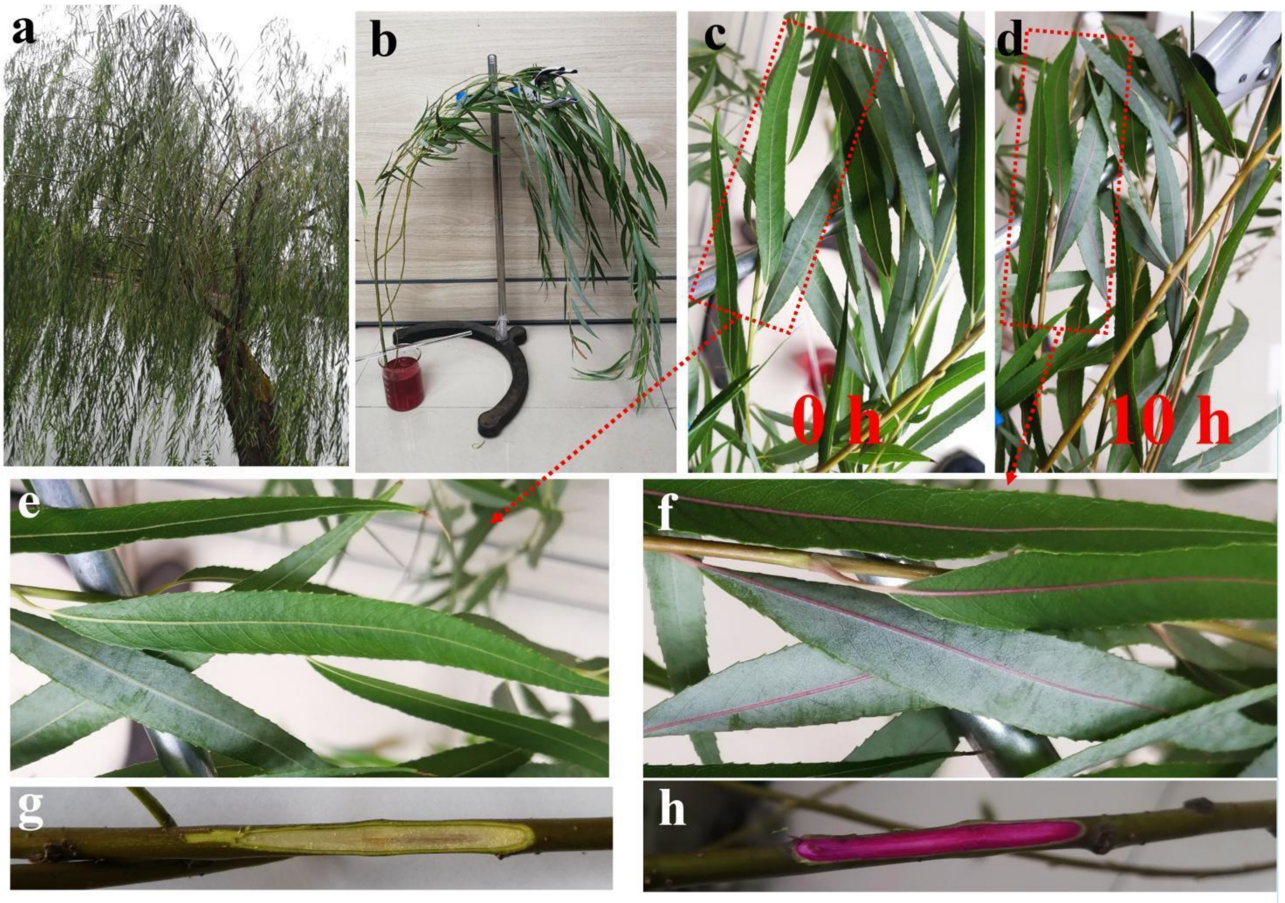

Fig. S1 (a) A willow growing near the water, (b) a bunch of willow branches inserted in rhodamine solution, (c, e) the original color of willow leaves, (d, f) leaves' color after the branches immersed in rhodamine for 10 h, (g) internal nature color of willow branch, (h) internal color of willow' branch immersed in rhodamine for 10 h.

The leaf vein of willow branches changed color from light white to pink within 10 h, illustrating the high water transport efficiency in willows.

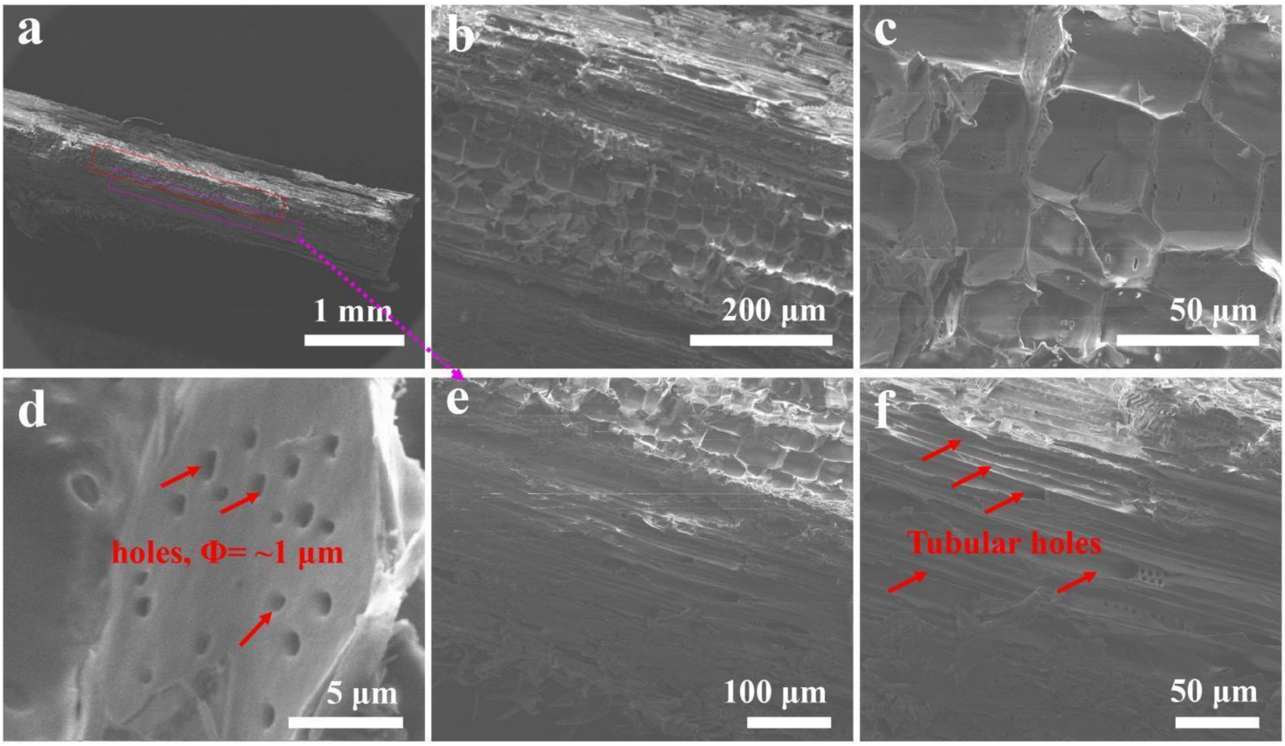

Fig. S2 SEM images of a willow branch. Large cell chambers were observed in the middle region (Fig. S2a, b, c), and many pores in the membrane of two adjacent cells were observed (Fig. S2d), water and nutrients can transport efficiently in those channels. Besides, numerous capillaries were observed (Fig. 2a, e, f), in which water and nutrients can transport efficiently in those capillaries.

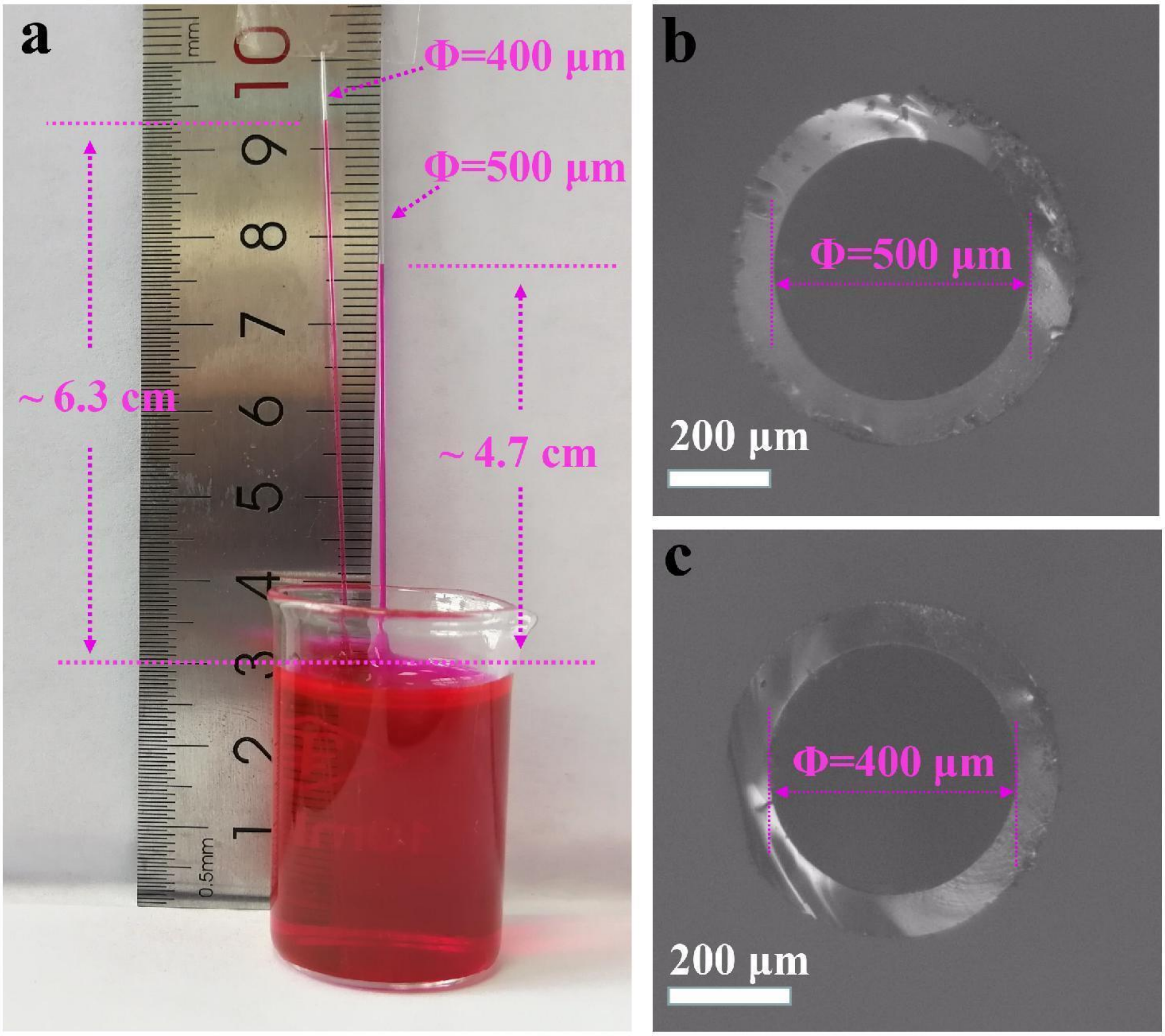

Fig. S3 (a) Digital image of water climbing in capillary tubes, liquid in the beaker is rhodamine aqueous solution, (b, c) optical microscope images of two capillaries with 500 and 400 μm diameter. The height of water climbing in the capillary can be calculated by the following equation:[7]

$$h = \frac{2\gamma}{\rho g r}$$

Where γ is the surface tension of water, 0.07214 N m^{-1} at 25 °C. ρ is the density of water, 997.043 kg m^{-3} at 25 °C. g is the earth's gravitational acceleration, about 9.806 m s^{-2}. r is the radius of the capillary. This experiment illustrates the smaller the radius curvature, the higher the additional pressure of the liquid subjected.

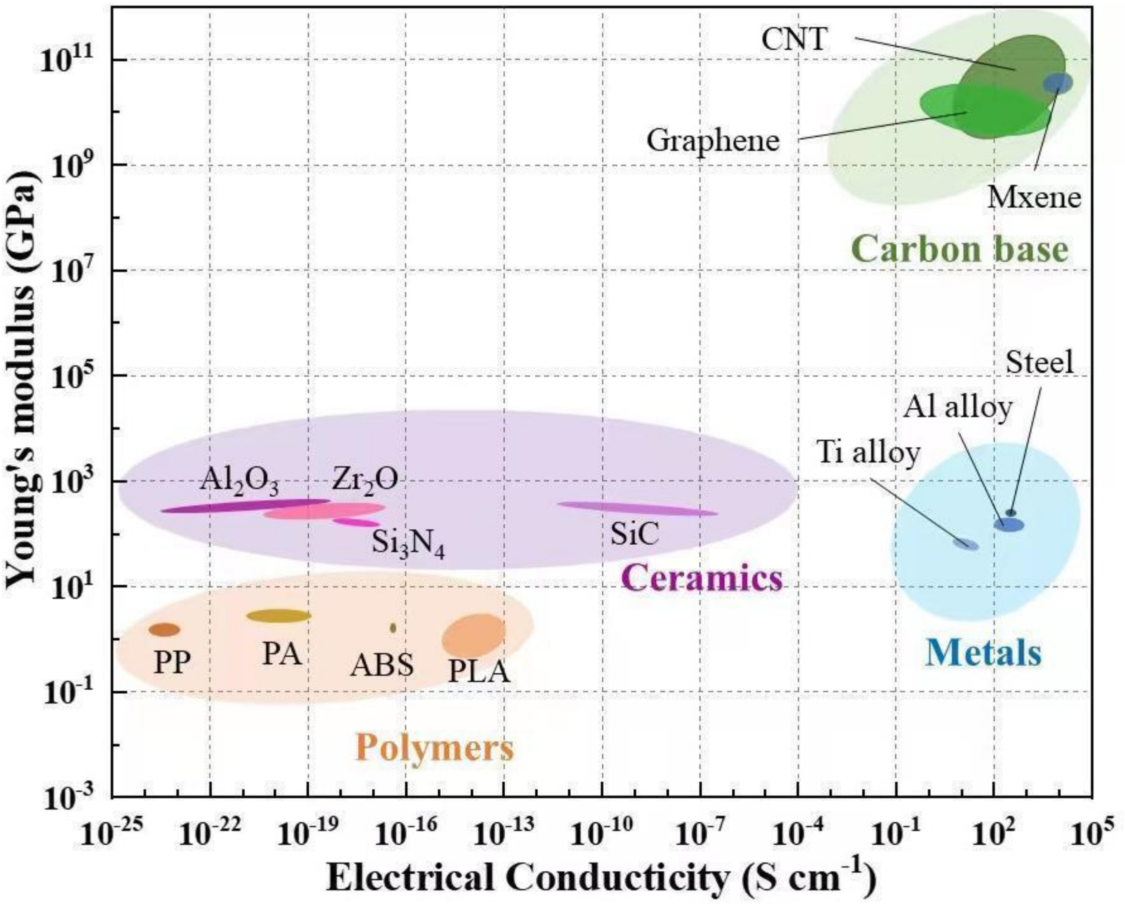

Fig. S4 Comparison of Young's modulus and electrical conductivity of graphene with a variety of materials.[8-10] The electrical conductivity of graphene is higher than polymer- and ceramic-type materials and reaches the same level as metals. However, Young's modulus of graphene is higher than that of metals. Note that the MXene and CNTs show the same conductivity level as graphene, however, MXene is expensive considering the large doses needed in DLP 3D printing, and the black CNTs need high UV-energy exposure density during DLP 3D printing processes, which is not suitable for ink with high curing content. In DLP 3D printing, utilizing inks with a high solid content of the conductive filler is essential for obtaining products with stable structures and high electronic conductivity. Graphene oxide possesses a brownish-yellow color, the ink containing graphene oxide can be photo-cured in low UV-energy exposure density, and graphene oxide can be reduced to graphene after heat treatment, thus graphene oxide was applied in this work.

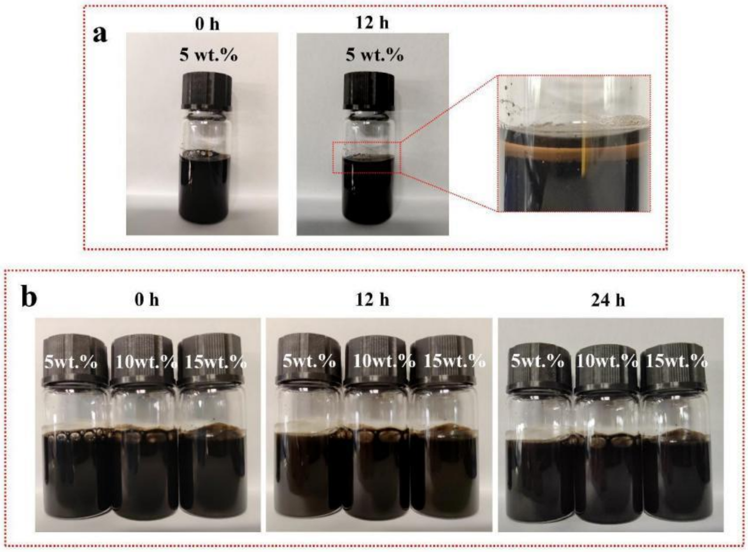

Fig. S5 Sedimentation experiment of graphite oxide (a) and fGO slurries (b).

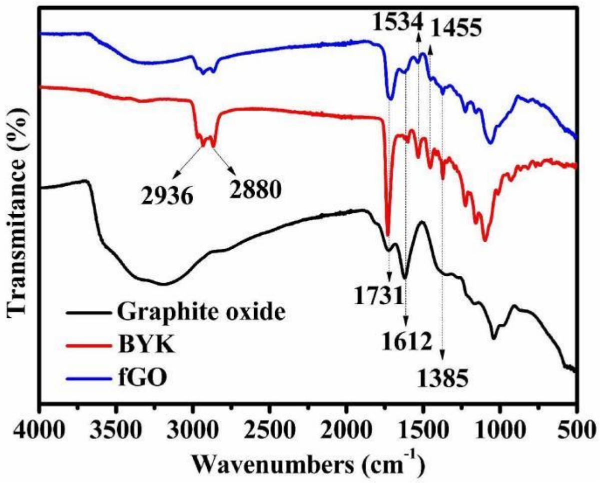

Fig. S6 FT-IR spectra of graphite oxide, BYK, and fGO.

The broad peaks at 1385 and 1455 cm^{-1} are the bending vibration peaks of C-H in methylene and C-H in methyl, and absorption peaks located at 1612 and 1731 cm^{-1} correspond to the stretching vibration of CH_2 in graphite oxide.[11-13] The absorption peaks located at 1534 cm^{-1} correspond to the bending vibration of the -NH group.[14, 15] Peaks at 2880 and 2936 cm^{-1} correspond to the characteristic peaks of methylene and methyl.[13, 16] According to the above analysis, -NH, $=CH_2$, and $-CH_3$ were anchored on the surface of graphite oxide after the functionalized by BYK.[17]

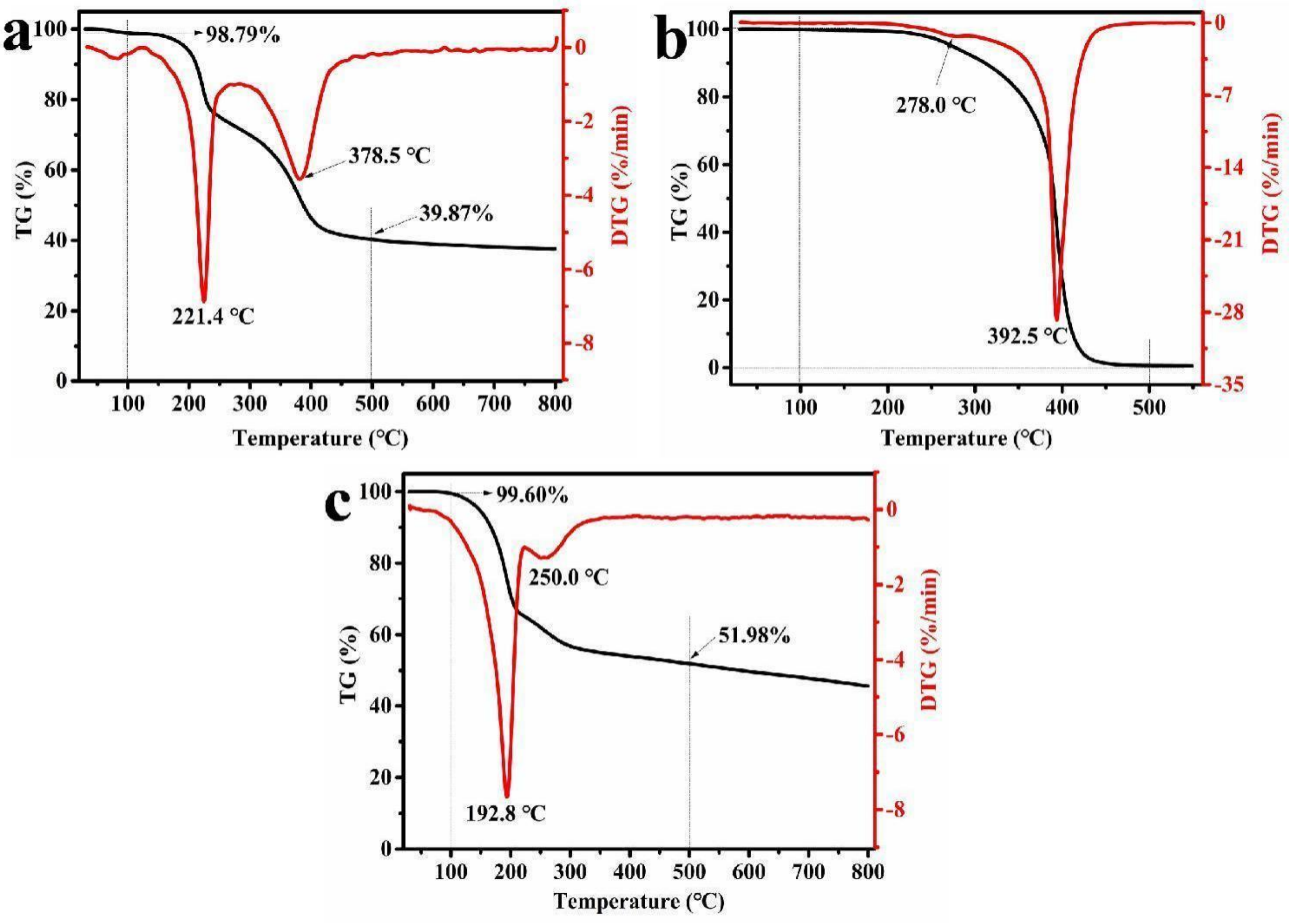

Fig. S7 TGA curves of graphite oxide (a), BYK (b), and fGO (c).

Graphite oxide showed a characteristic weight-loss peak at 192.8 °C, and BYK showed a characteristic weight-loss peak at 392.5 °C, respectively. The weight loss of fGO in 198.2 °C and 250.0 °C were originated from the pyrolysis of oxygen-containing functional groups and the BYK inside the fGO. The BYK content in fGO can be calculated according to weight loss. When the temperature is below 100 °C, slight weight loss was observed in graphite oxide and fGO, which were caused by the volatilization of adsorbed water. No obvious weight loss was observed in BYK below 100 °C. When the temperature exceed 500 °C, almost all the BYK was decomposed. The BYK content was calculated to 11.3 wt.% according to weight loss [(51.98 wt.% - 39.87 wt.%) - (99.60 wt.% -98.79 wt.%)].

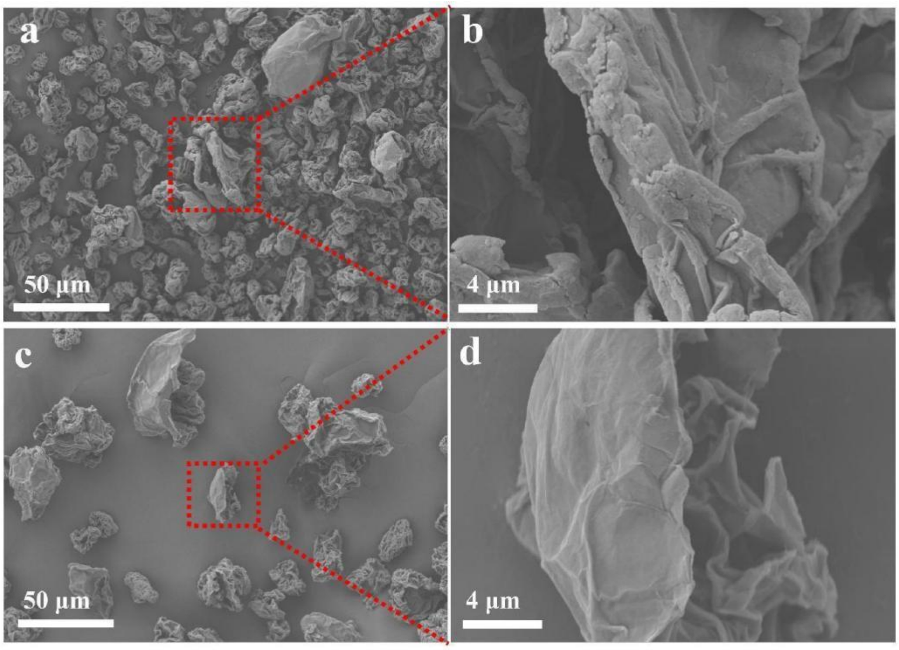

Fig. S8 SEM images of commercial graphite oxide (a, b) and fGO (c, d). Lots of curls and wrinkles were observed in graphite oxide and fGO.

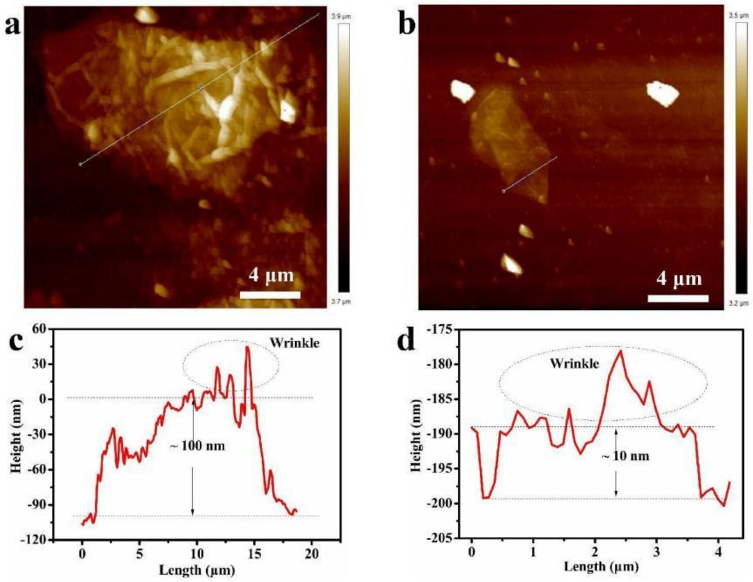

Fig. S9 Atomic force microscope images of graphite oxide (a) and fGO (b), the height profile of graphite oxide (c) and fGO (d).

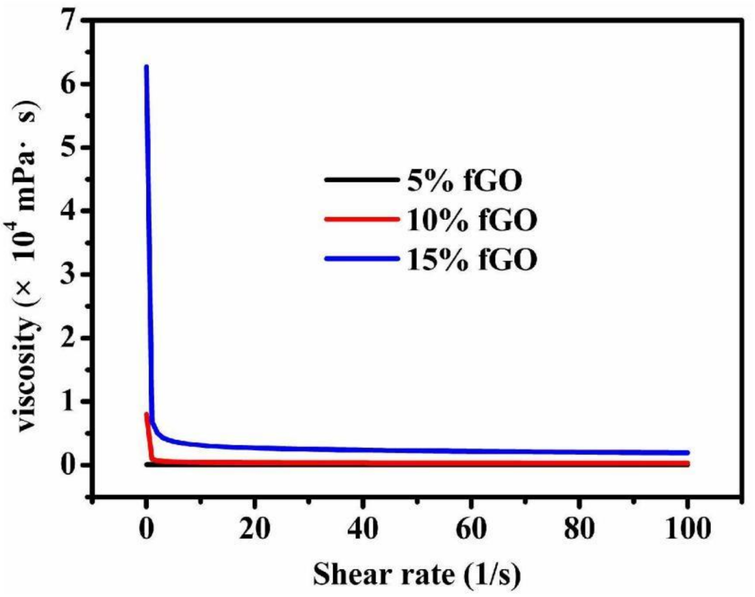

Fig. S10 Shear thinning property of ink with various fGO content.

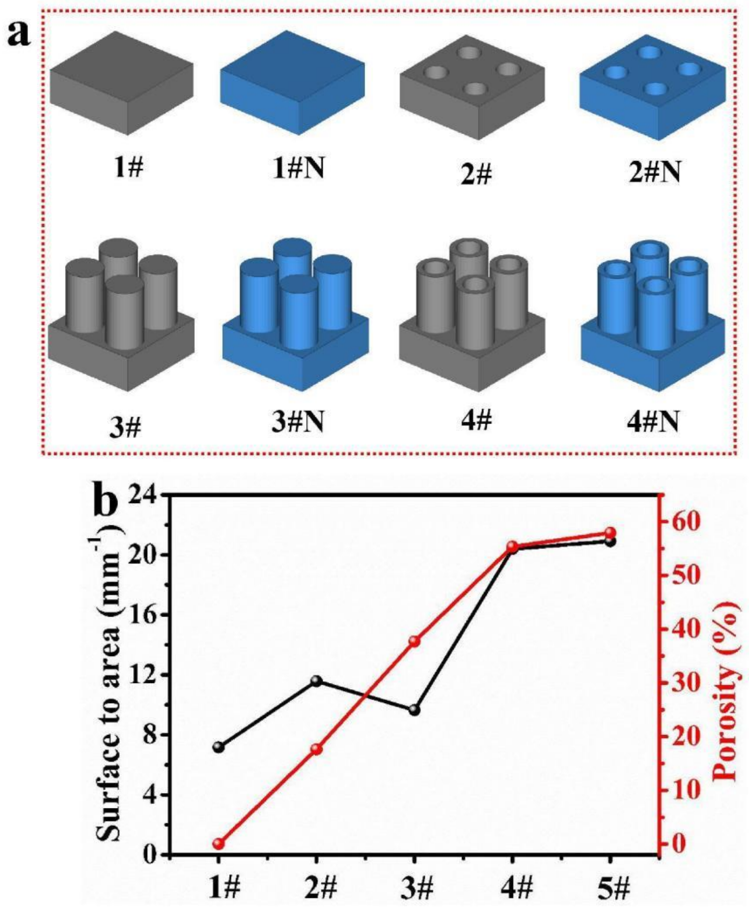

Fig. S11 (a) Models of 1#, 2#, 3#, 4#, 1#N, 2#N, 3#N and 4#N and (b) surface to area and porosity of *n*# (*n*=1, 2, 3, 4, 5).

Table S1 Structural parameters of printed catalyzer supports. The unit of numbers: mm.

No.	Length	Width	High	Supplementary notes	Height of columns or tubes
1#	8	8	0.3	Slab	/
2#	8	8	0.3	Slab with holes array. Holes diameter: 0.2, Distance between two Holes' centers: 0.4	/
3#	8	8	0.8	Columns array on the slab. Columns diameter: 0.3, Distance between two columns: 0.4	0.5
4#	8	8	0.8	Tubes array on the slab. Inner diameter: 0.2, Outside diameter: 0.3, no side holes	0.5
5#	8	8	0.8	Tubes array on the slab. Inner diameter: 0.2, Outside diameter: 0.3, Diameter of side holes: 0.15	0.5

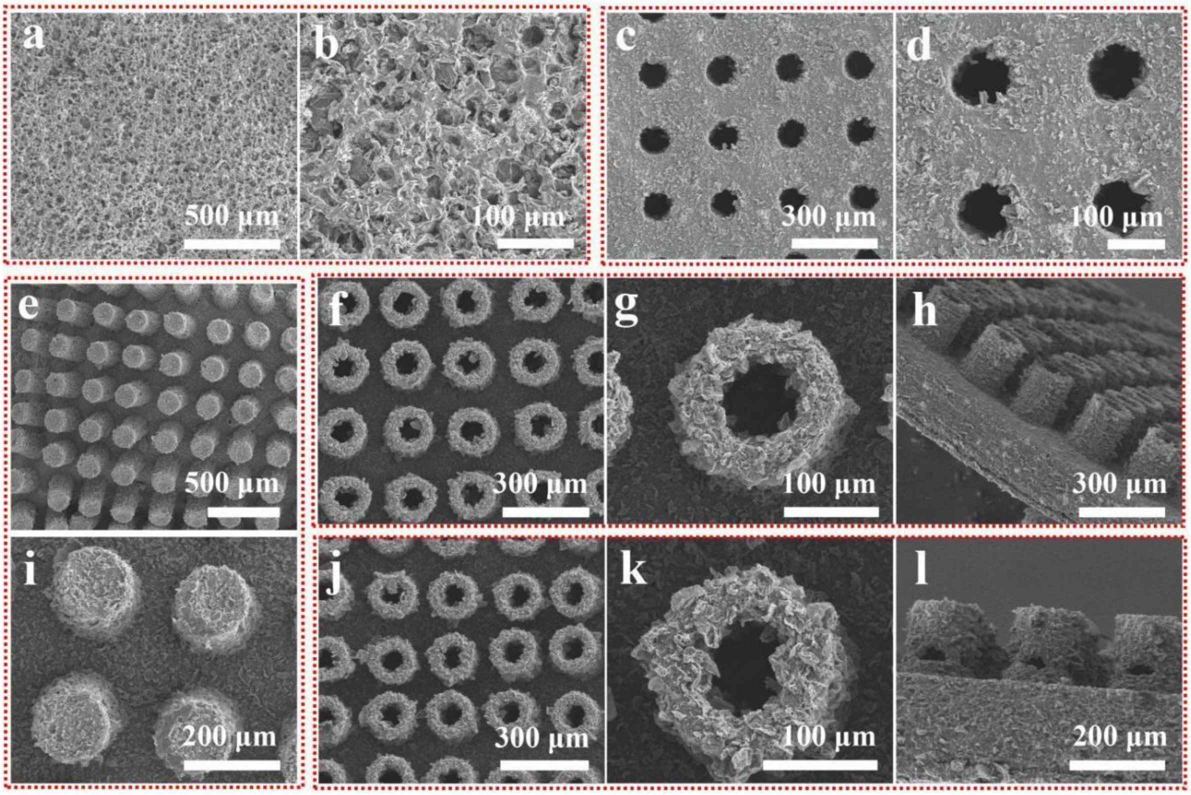

Fig. S12 SEM images of 1# (a, b), 2# (c, d), 3# (e, i), 4# (f, g, h), and 5# (j, k, l).

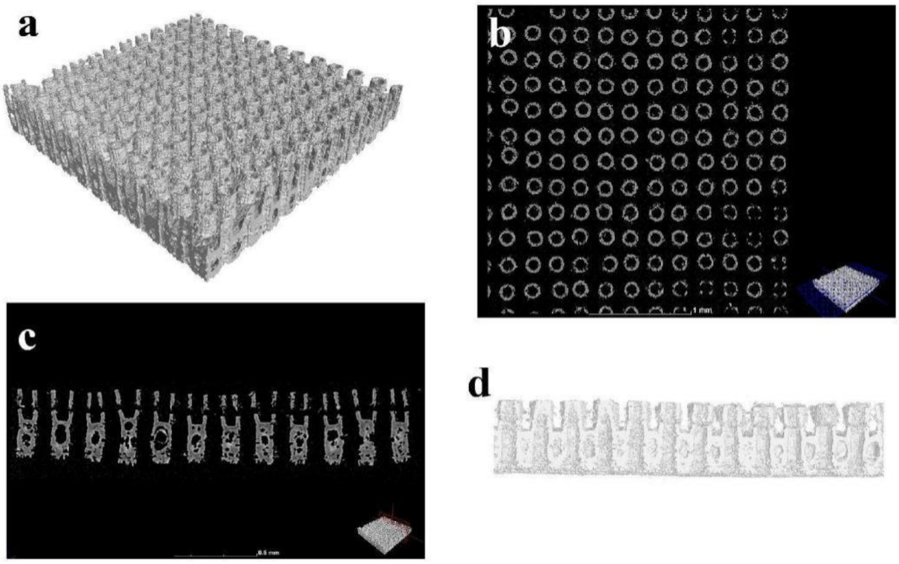

Fig. S13 Three-dimensional computed tomography images of 5#. Side holes on capillaries were seen clearly in figure c and d.

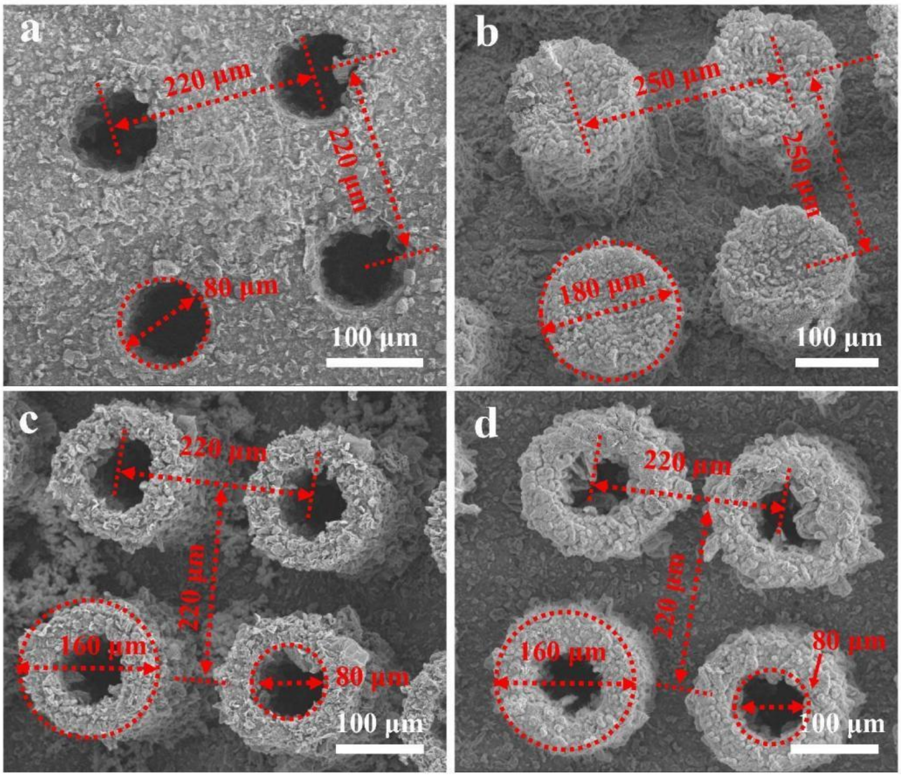

Fig. S14 The structural parameters of 2# (a), 3# (b), 4# (c), and 5# (d) from SEM images.

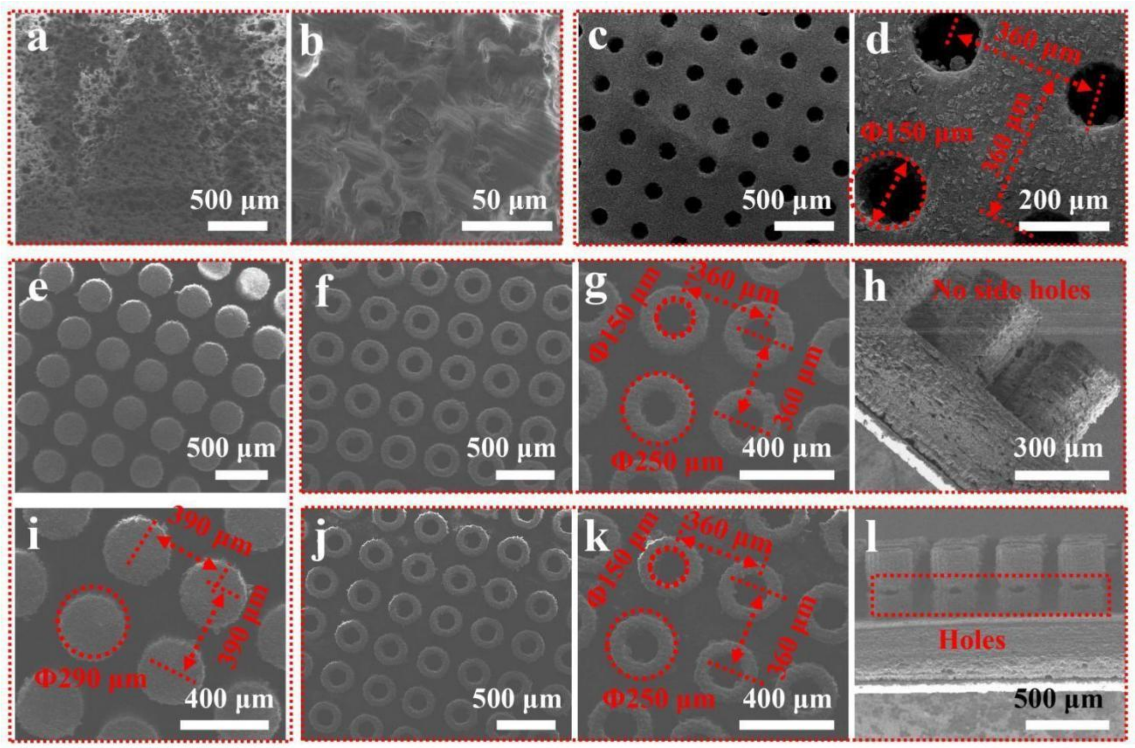

Fig. S15 SEM images of newly printed 1# (a, b), 2# (c, d), 3# (e, i), 4# (f, g, h), and 5# (j, k, l) without heated treatment.

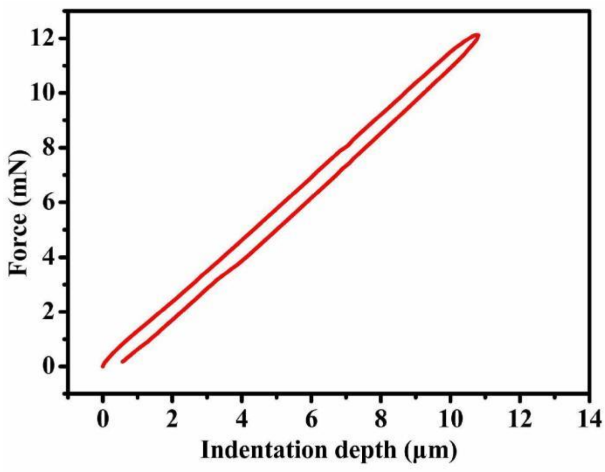

Fig. S16 Nanoindentation curve of 1#.

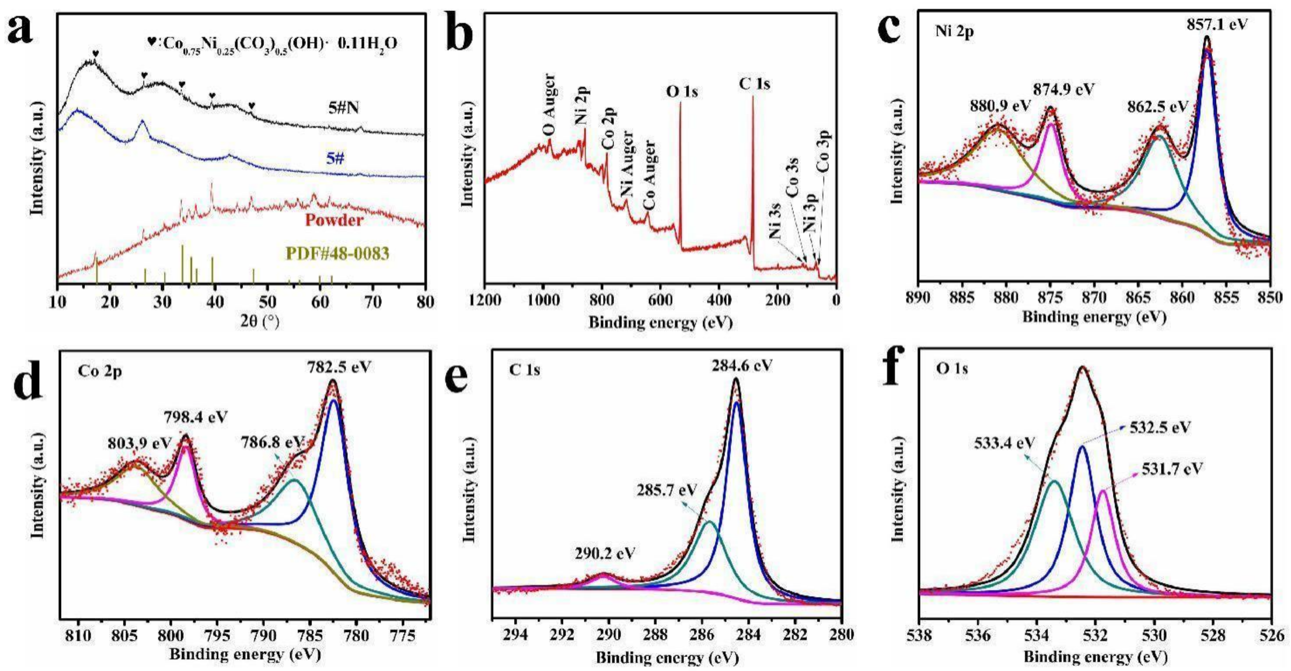

Fig. S17 XRD curves of CoNiCH powder, 5# and 5#N (a), XPS survey of CoNiCH powder (b), Ni 2p (c), Co 2p (d), C 1s (e), and O 1s (f) spectra of CoNiCH powder.

According to previous research, a one-step hydrothermal of the Co^{2+}, Ni^{2+} solution by using $CO(NH_2)_2$ as the precipitation agent will produce Ni-substituted cobalt carbonate hydroxide.[18-22] In this work, the catalyst grew on *n*# was fabricated by one-step hydrothermal of the $NiCl\cdot6H_2O/CoCl\cdot6H_2O/CO(NH_2)_2$ solution, the catalyst should be Ni substituted cobalt carbonate hydroxide, $Co_{0.75}Ni_{0.25}(CO_3)_{0.5}(OH)\cdot0.11H_2O$.

The 5# showed three broad diffraction peaks at 13.7°, 26.2°, and 42.7° in the XRD curve, which

were also observed in 5#N. Those peaks originated from graphene/carbon. Besides the graphene/carbon peaks, others can be matched to standard diffraction peaks of PDF#48-0083, an orthorhombic cobalt carbonate hydroxide hydrate, and no other impurity peaks, proving the Ni atoms substitute the Co atoms in the $Co(CO_3)_{0.5}(OH)\cdot0.11H_2O$ lattice (CoNiCH).[18, 20, 22] The diffraction peaks of CoNiCH were also observed in 5#N, indicating CoNiCH existed in 5#N. In XPS survey spectra, O, Ni, Co, and C elements were detected. O elements may originate from residual oxygen-containing functional groups, or a trace of H_2O/CO_2 was adsorbed on the surface during the test.[22-24] The peaks at 857.1 and 874.9 eV in the Ni 2p spectrum were attributed to Ni $2p_{3/2}$ and Ni $2p_{1/2}$, and two satellite peaks observed at 862.5 and 880.9 eV were the Ni^{2+} in the CoNiCH.[18, 20, 21] The Co 2p spectrum was fitted and divided into Co $2p_{3/2}$ at 782.5 eV and Co $2p_{1/2}$ at 798.4 eV, and two satellite peaks of Co^{2+} at 786.8 and 803.9 eV.[18-20, 22, 25, 26] The C 1s signals can be well-fitted to three peaks located at 284.6 eV for C-C, 285.7 eV for C-O, and 286.6 eV for C=O.[18-20, 22, 27] The O 1s can be fitted to three peaks located at 531.7 eV for Co-O/Ni-O, 532.5 eV for C-O, and 533.4 eV for C=O bonds.[19, 20, 22, 28]

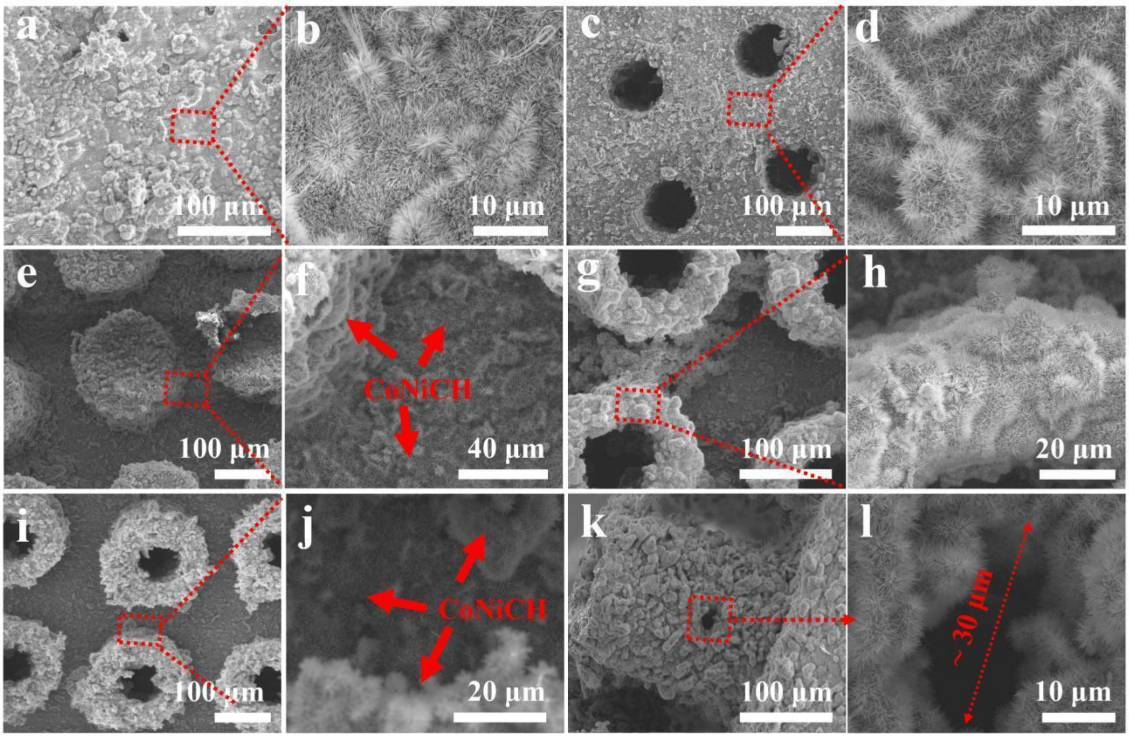

Fig. S18 SEM images of 1#N (a, b), 2#N (c, d), 3#N (e, f), 4#N (g, h, i), and 5#N (j, k, l). Those SEM images revealed all surfaces of catalytic supports were covered by CoNiCH needles.

Table S2 The content of CoNiCH catalyst loading on *n*#N and Ni foam. The CoNiCH catalyst grown on Ni foam was denoted as CoNiCH/Ni in the main text.

Sample number	A brief description	Loading of CoNiCH (mg cm^{-2})
1#N	1# coated with CoNiCH	3.74
2#N	2# coated with CoNiCH	4.37
3#N	3# coated with CoNiCH	4.35
4#N	4# coated with CoNiCH	4.46
5#N	5# coated with CoNiCH	4.13
CoNiCH/Ni	Ni foam coated with CoNiCH	4.95

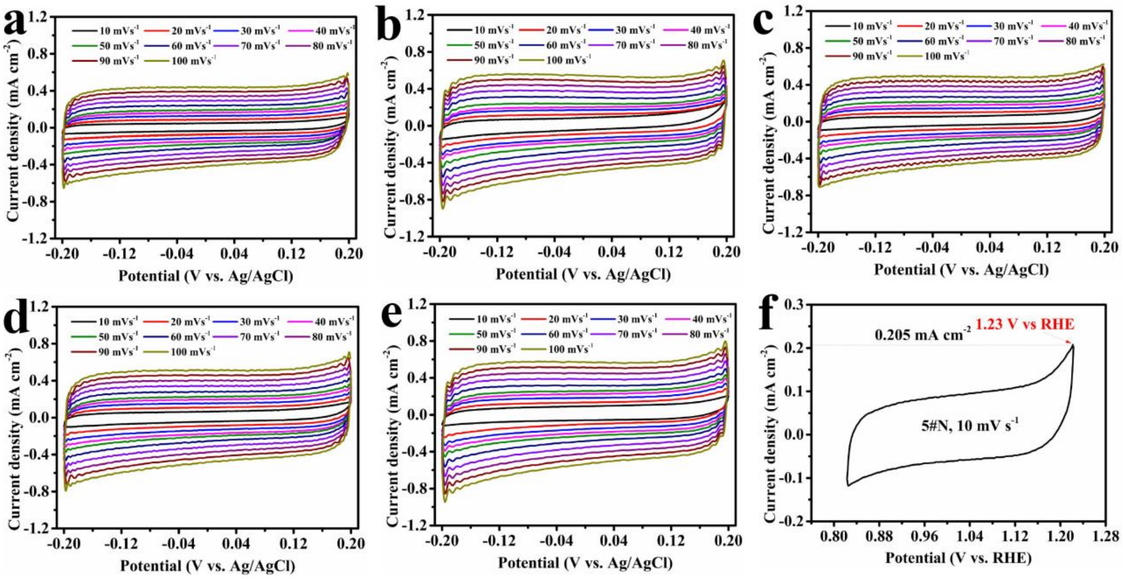

Fig. S19 CV curves of OER scanned on -0.2~0.2 V vs. Ag/AgCl. (a) 1#N, (b) 2#N, (c) 3#N, (d) 4#N, and (e) 5#N. In this test, the *n*#N was the working electrode, the Pt electrode was the counter electrode, and the Ag/AgCl electrode was the reference electrode, (f) CV curve of 5#N scanned on 10 mVs^{-1} converted to standard hydrogen electrode potential as the reference potential.

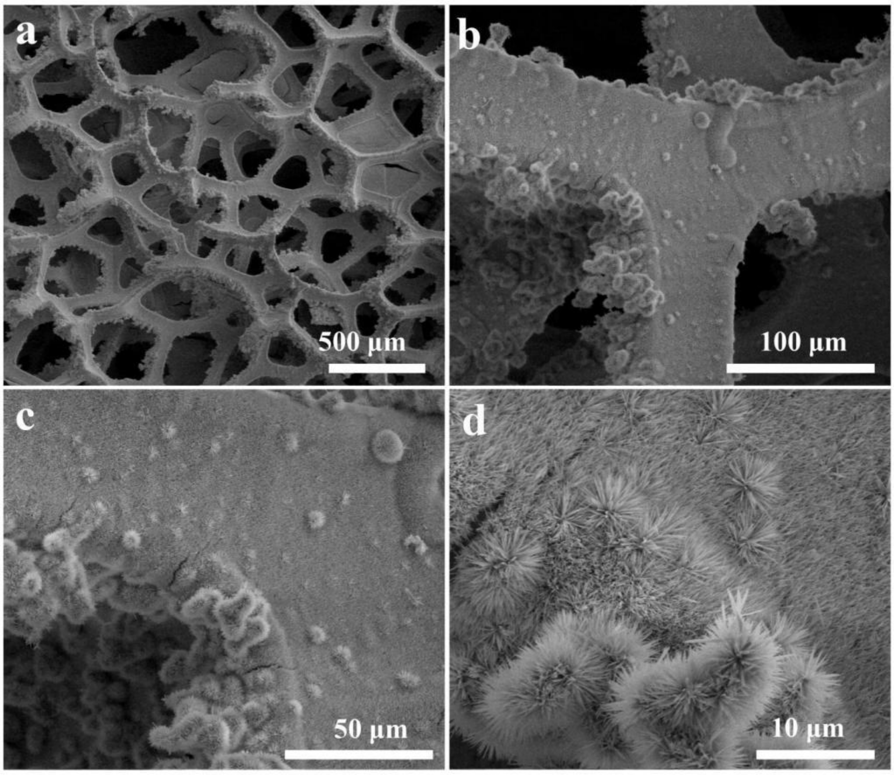

Fig. S20 SEM images of CoNiCH planted Ni foam (CoNiCH/Ni).

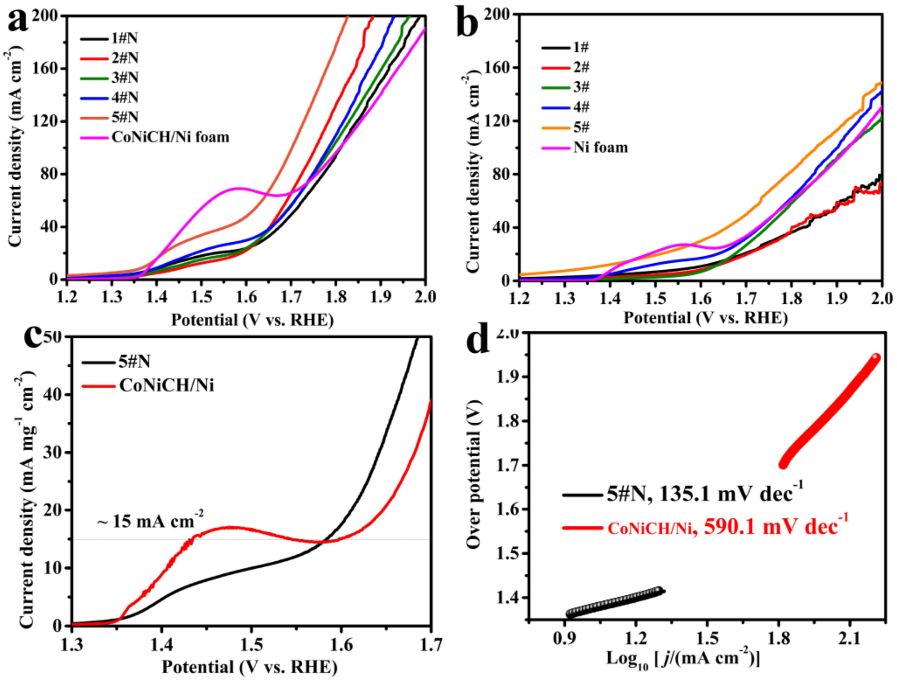

Fig. S21 (a) LSV without IR compensation of *n*#N (*n*=1~5) and CoNiCH/Ni scanned on 10 mV s^{-1} in a three-electrodes system, (b) LSV without IR compensation of *n*# (*n*=1~5) and Ni foam scanned on 10 mV s^{-1} in a three-electrodes system, (c) compare the LSV curve of 5#N and CoNiCH/Ni normalized on the weight of CoNiCH loading, (d) Tafel slope of 5#N and CoNiCH/Ni calculated

from LSV curves of the figure (a).

In Fig. S21a, the oxidation peak of CoNiCH/Ni around 1.5 V originated from Ni oxidation, the Ni oxidation peak was also observed in Ni foam, as shown in Fig. S21b. [29,30] Thus, the Tafel slope of CoNiCH/Ni should be calculated in the voltage of above 1.65 V. The Tafel slope of 5#N and CoNiCH/Ni were 135.1 mV dec^{-1} and 590.1 mV dec^{-1}, respectively.

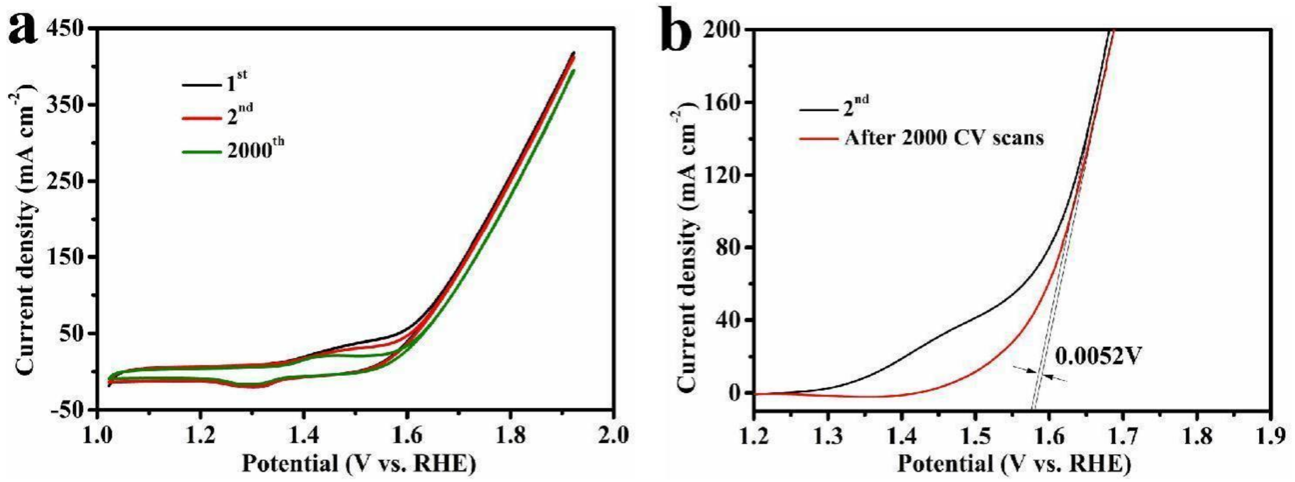

Fig. S22 Long-team CV cycles of 5#N. (a) the 1st, 2nd and 2000th CV curves scanned in 0 ~ 0.9 V at a three-electrodes system, (b) compare the cathode scan of the 2nd and 2001st cycle. The reproduction of the curves' profile illustrated the stability of 5#N.

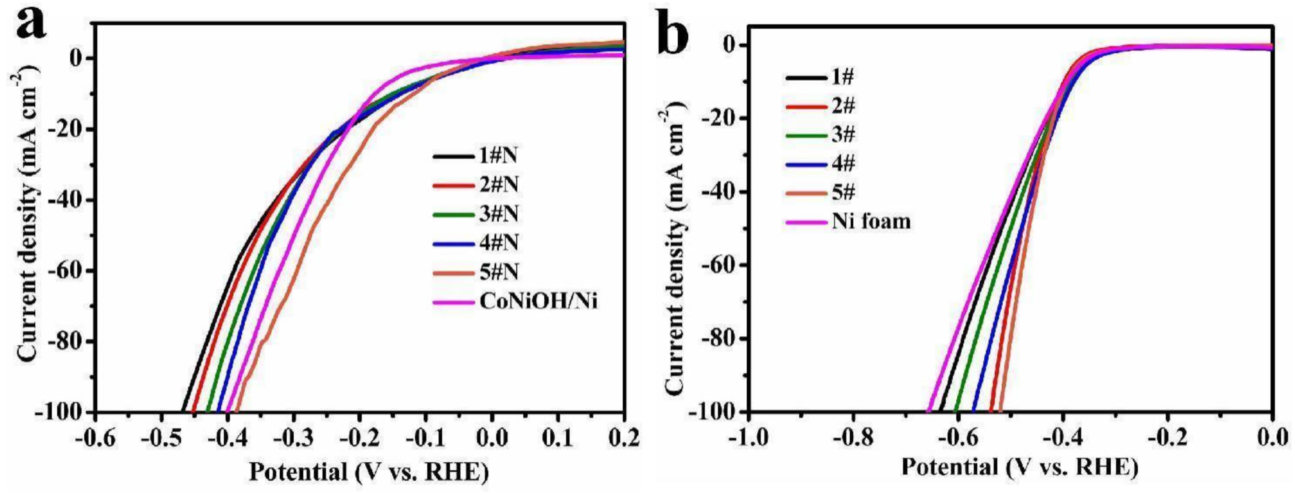

Fig. S23 (a) LSV curves of HER without IR compensation. (a) n#N and CoNiOH/Ni and (b) *n*# and Ni foam.

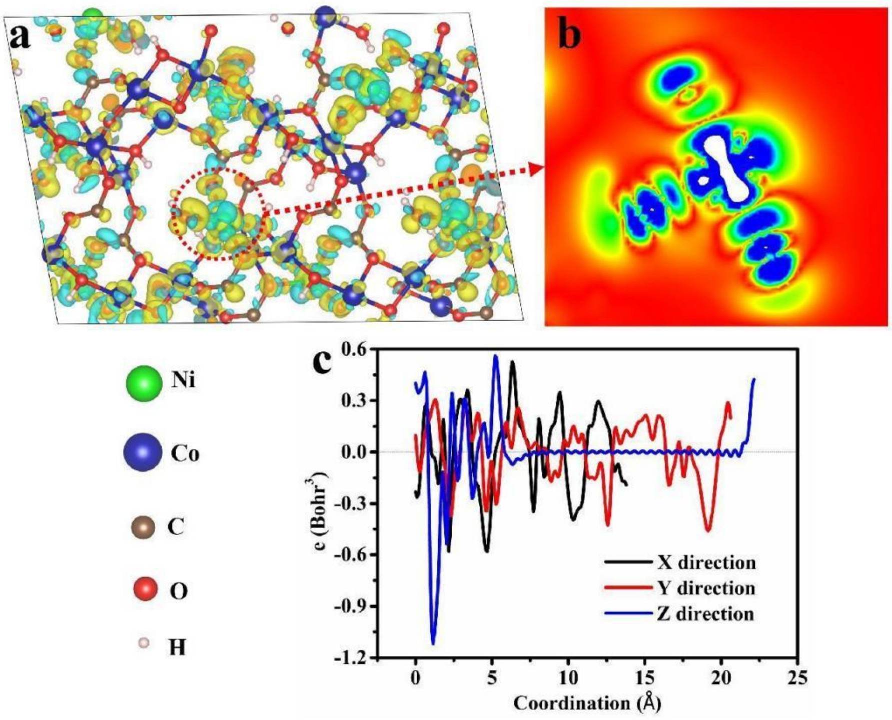

Fig. S24 (a) Charge density difference of Ni substituted $Co(CO_3)_{0.5}(OH)$, (b) electron cloud overlap of Ni atom with O, C, H atoms in $Co_{0.75}Ni_{0.25}(CO_3)_{0.5}(OH)$, (c) electron density fluctuation of a Ni atom in X, Y, Z directions.

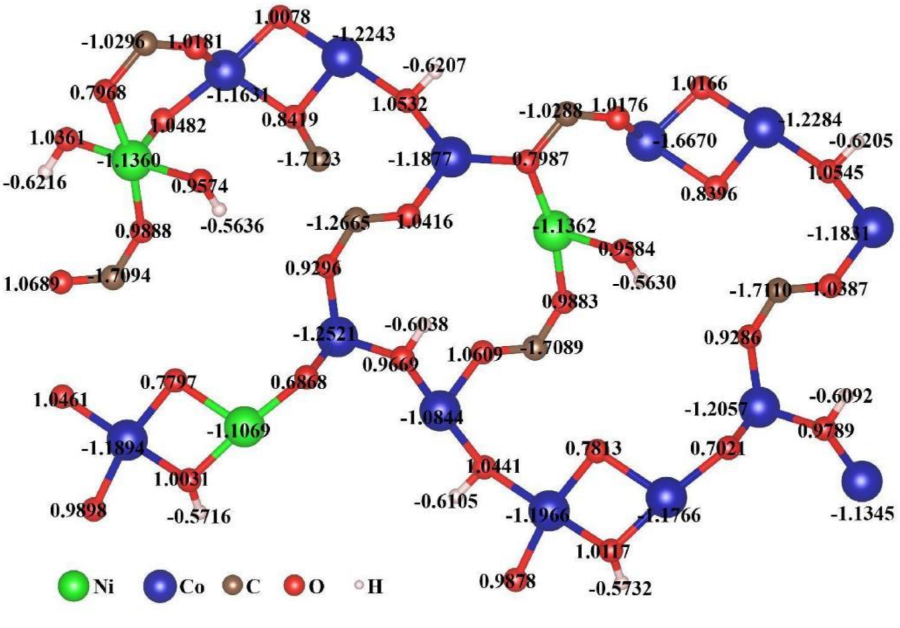

Fig. S25 Bader charges analysis of (1 2 1) interface on $Co_{0.75}Ni_{0.25}(CO_3)_{0.5}(OH)$.

The Co, Ni, C, and H atoms lose valence electrons and bader charge values are negative, tend to adsorb negative charged group/atom, and therefore are potential OER active sites, while O atoms get electrons and show positive values, thus are HER active sites.

Supplementary Text 1

The H_2O split is a four-electron reaction process, the process can be written by the following equations:[31, 32]

$$\text{Step1:} \quad ^{*} + OH^{-} \rightarrow OH^{*} + e^{-}$$

$$\text{Step2:} \quad OH^{*} + OH^{-} \rightarrow O^{*} + H_2O + e^{-}$$

$$\text{Step3:} \quad O^{*} + OH^{-} \rightarrow OOH^{*} + e^{-}$$

$$\text{Step4:} \quad OOH^{*} + OH^{-} \rightarrow O_2 + {}^{*} + H_2O$$

Where * represents the active sites of CoNiCH, that are Ni, Co, C, and H atoms of (1 2 1) plane, OH^{-} is hydroxyl, OH^{*} is oxyhydrogen radical adsorb on active site, O^{*} is oxygen radical adsorb on the active site, OOH^{*} is superoxide radical adsorb on active site. The Gibbs free energy (△G) of each step can be calculated based on the following equations:[31, 32]

$$\Delta G_1 = G(OH^{*}) - G(^{*}) - G(OH^{-})$$

$$\Delta G_2 = G(O^{*}) + G(H_2O) - G(OH^{*}) - G(OH^{-})$$

$$\Delta G_3 = \Delta G(OOH^{*}) - G(O^{*}) - G(OH^{-})$$

$$\Delta G_4 = 4.92 - \Delta G1 - \Delta G2 - \Delta G3$$

The △G values of the C and H sites are listed in Table S3. The ΔG_1 of the C site is -4.75 eV, illustrating the strong adsorb of OH^{-} in the C site, and such strong adsorb leads to the C not being suitable for the active site of OER. In the H site situation, the ΔG2 reached -3.46 V, illustrating the strong chemical bond between * and -OH, thus H was not suitable for the active site of OER.

Table S3 △G of H_2O split on C and H sites. Energy unit: eV

Elements	Step-1	Step-2	Step-3	Step-4
C	-4.75	2.51	2.43	4.73
H	-0.99	-3.46	6.62	2.75

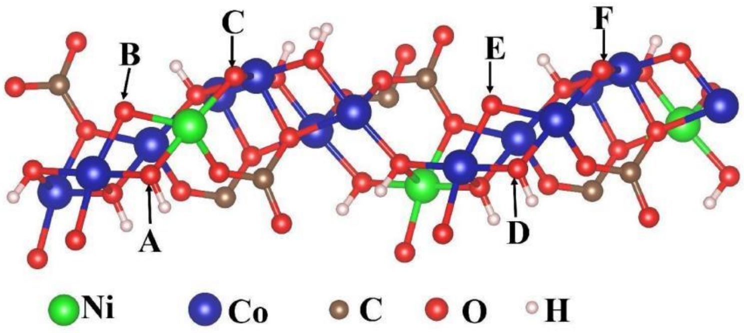

Fig. S26 Denote the O atoms surrounding Ni and Co. Only the protuberant O atoms (denoted to A-F) are calculated in the △G of hydrogen adsorption. The HER energy on those O sites is listed in Table S4.

Table S4 H adsorb $\triangle G_{H*}$ on O denoted in Fig. S26.

A	B	C	D	E	F
-0.1461	-0.0609	-0.5135	0.494	-0.2439	-0.5949

The $\triangle G_{H*}$ was calculated based on follow equation:[33]

$$\triangle G_{H*} = E_{ads}(H^*) + 0.24\ \mathrm{eV}$$

$$E_{ads}(H^*) = E_{based\text{-}H} - (E_{based} + E_H)$$

Where $E_{ads}(H^*)$ is the adsorbed energy of the H atom, $E_{based\text{-}H}$ is the total energy of H adsorbed on based, E_{based} is the energy of based, and E_H is the energy of the H atom. The positive value of $\triangle G_{H*}$ implies it was not suitable for the active site of HER, and $|E_{ads}(H^*)|$ close to 0 implies it was more active for HER. The E site is not considered for HER active site due to the positive of $\triangle G_{H*}$. The $|E_{ads}(H^*)$, A| and $|E_{ads}(H^*)$, B| are less than $|E_{ads}(H^*)$, E|, and $|E_{ads}(H^*)$, C| is less than $|E_{ads}(H^*)$, F|, illustrate O surround Ni is more active than that of surround Co for HER.

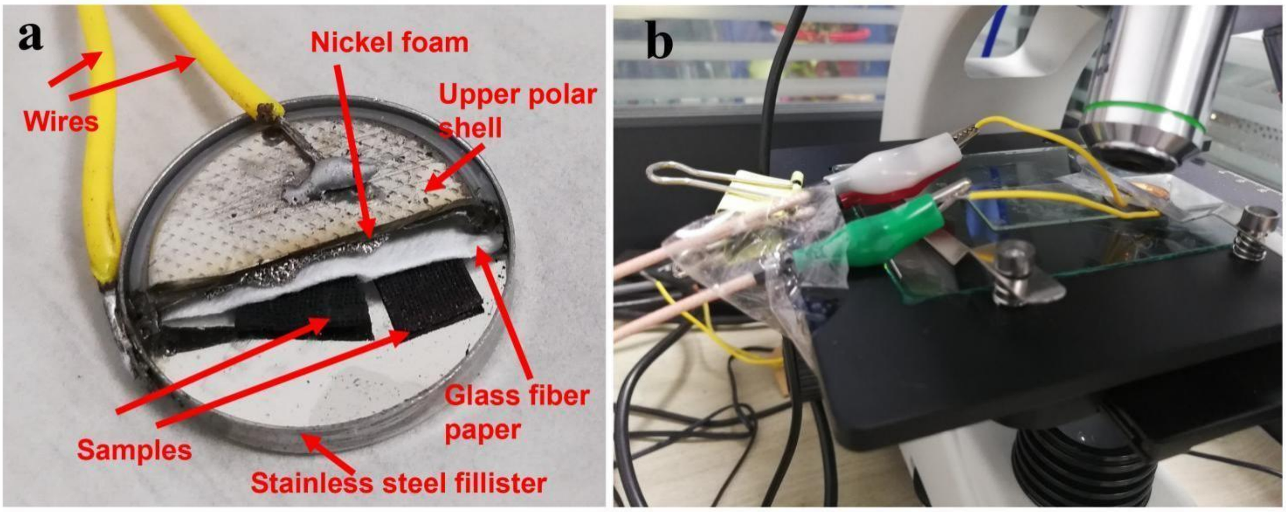

Fig. S27 A cell designed for in-situ optical microscope observation of bubbling in front view. (a) the stacking order of parts, (b) the wires connected with the cell. In OER, the positive electrode connected with the stainless steel fillister, and the negative electrode connected with the upper polar shell, this connection was contrary to HER observation.

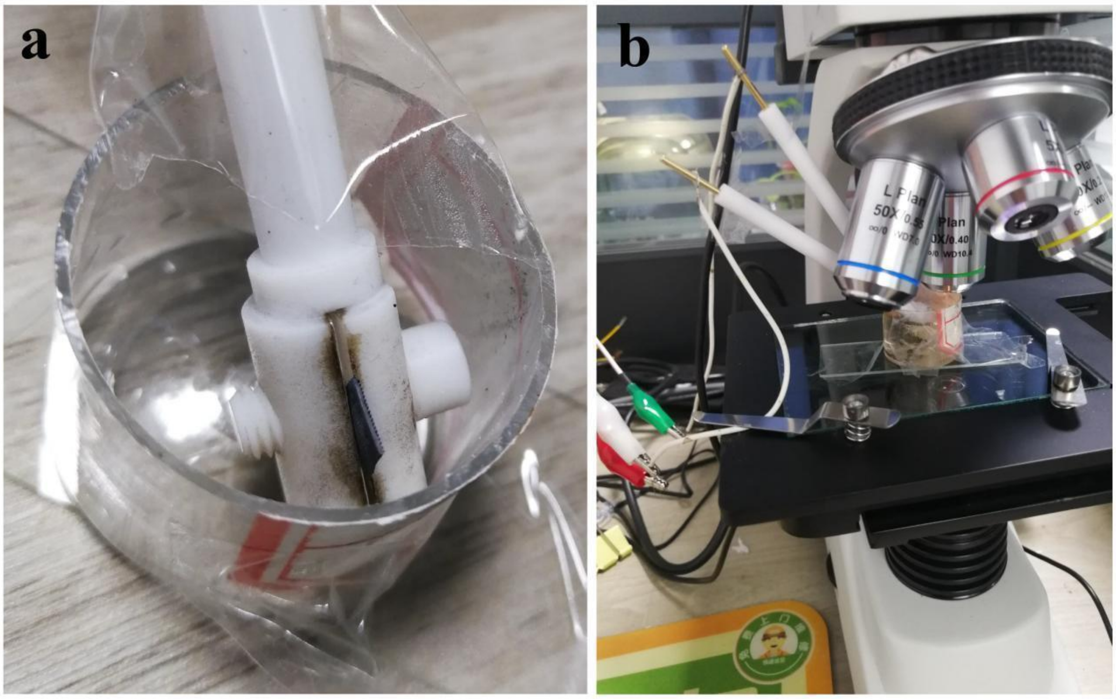

Fig. S28 A cell designed by using a polypropylene plastic bottle for in situ optical microscope observation of bubble release from side holes. (a) spatial positioning image of 5#N, (b) the wire connected with the cell. In OER, the positive electrode connected with the sample, and the negative electrode connected with the Pt electrode, this connection was contrary to HER observation.

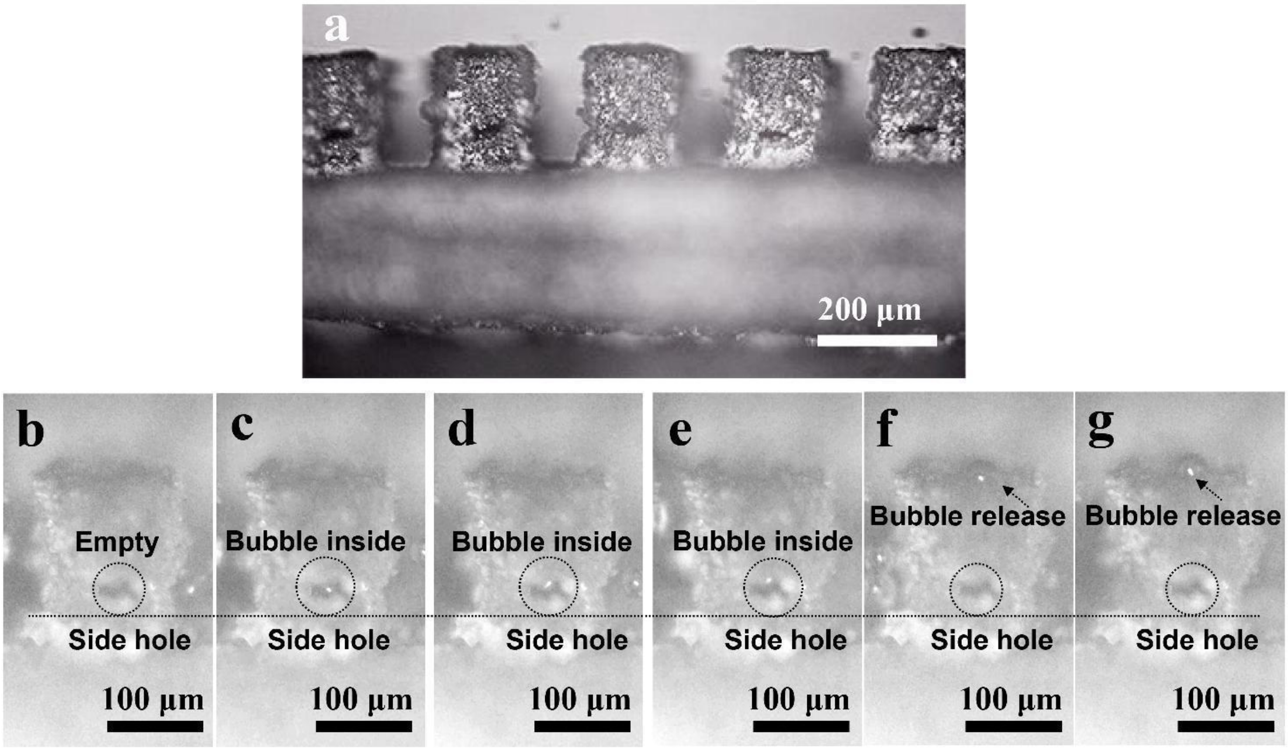

Fig. S29 (a) Side view of 5#N observed in an optical microscope, (b-e) the H_2 release from the orifice of capillary in 1 mol L^{-1} NaOH.

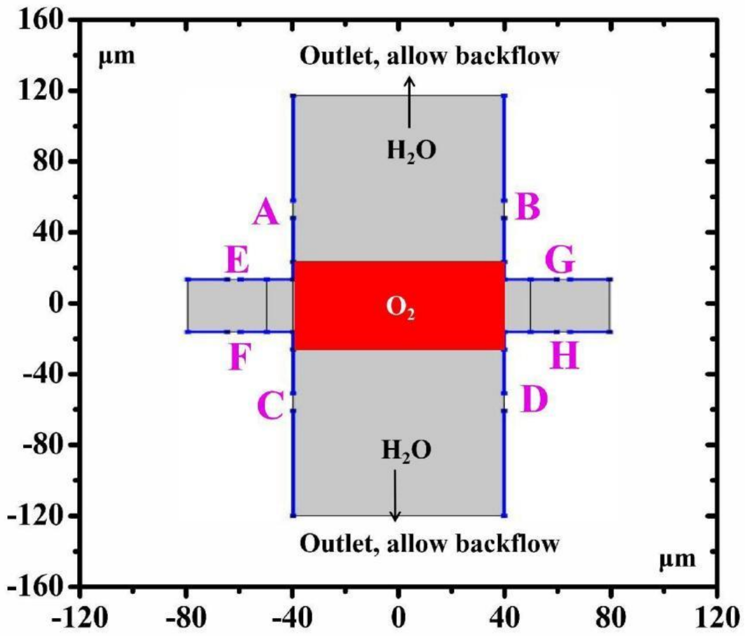

Fig. S30 A two-dimensional model of capillary for H_2O flow/O_2 release simulation. A-H marked the bubble occurring points.

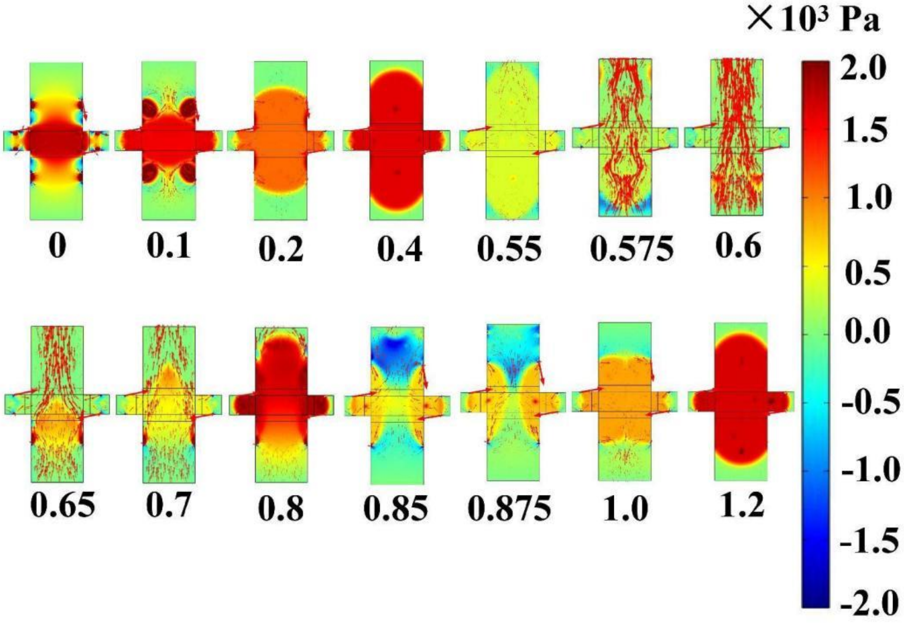

Fig. S31 Pressure simulation in a 1.2 ms period.

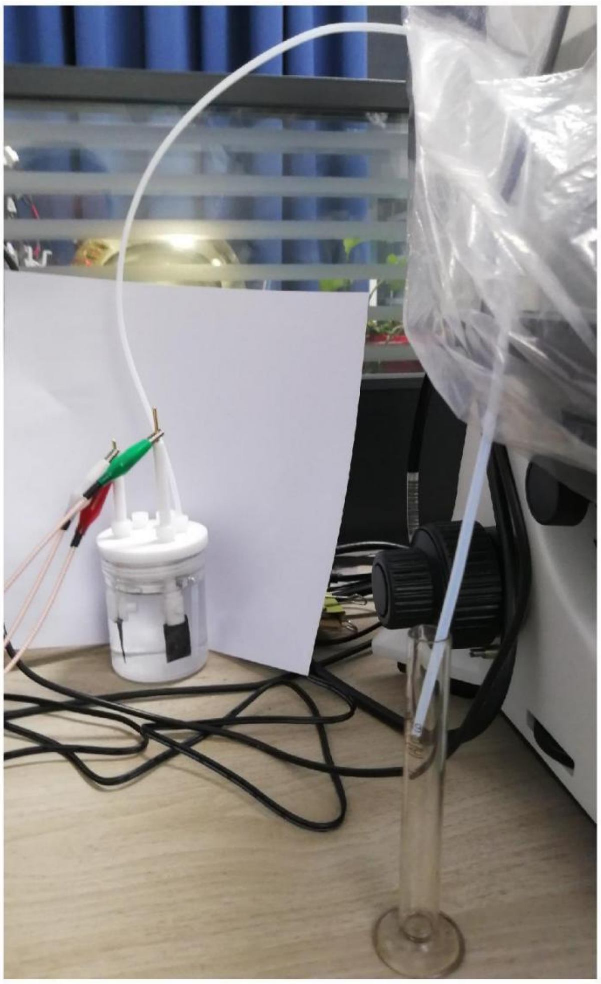

Fig. S32 Bird view of the device for measuring the gas volume by drainage method during overall water splitting.

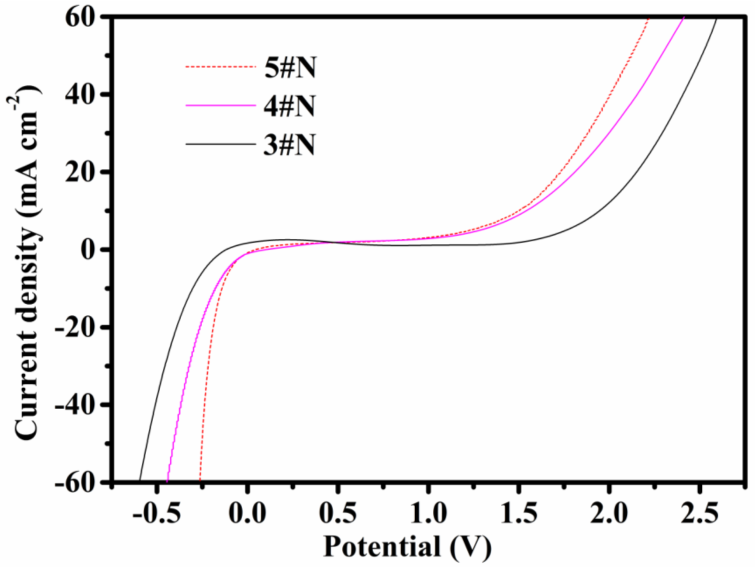

Fig. S33 LSV profile of 3#N, 4#N, 5#N without IR compensation. The LSV was tested in a two-electrodes cell, the curve of 5#N is the same as Fig. 5e of the main text, only for guiding the eyes in this figure to compare the overall water splitting performances of 3#N, 4#N, and 5#N.

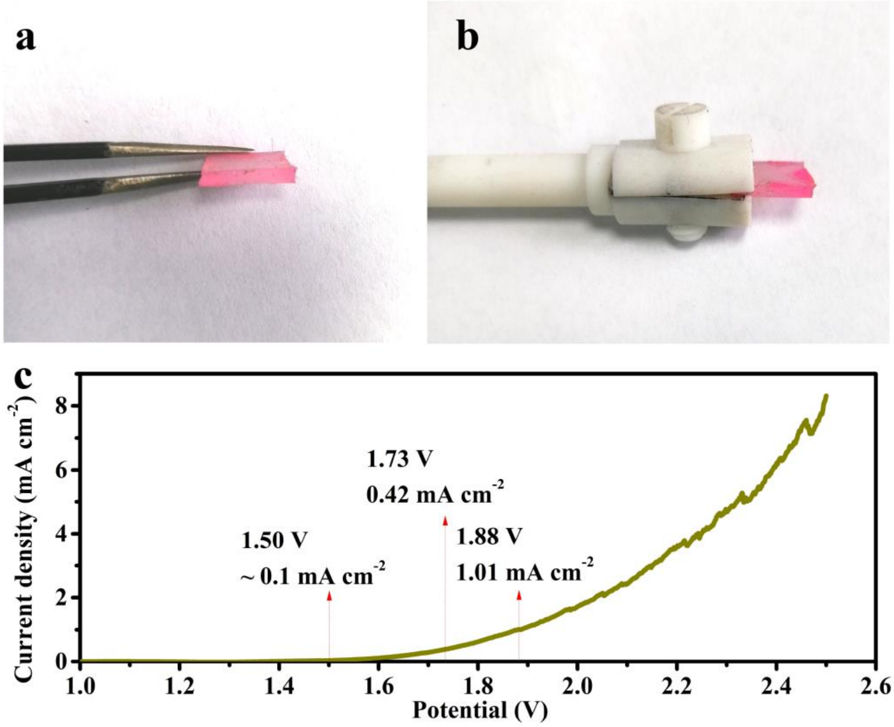

Fig. S34 (a) A plastic disc with the same thickness of 5#N, (b) a plastic disc clamped by a Pt electrode, (c) the LSV curve of Pt/Pt electrode scanned at 10 mV s^{-1} without IR compensation.

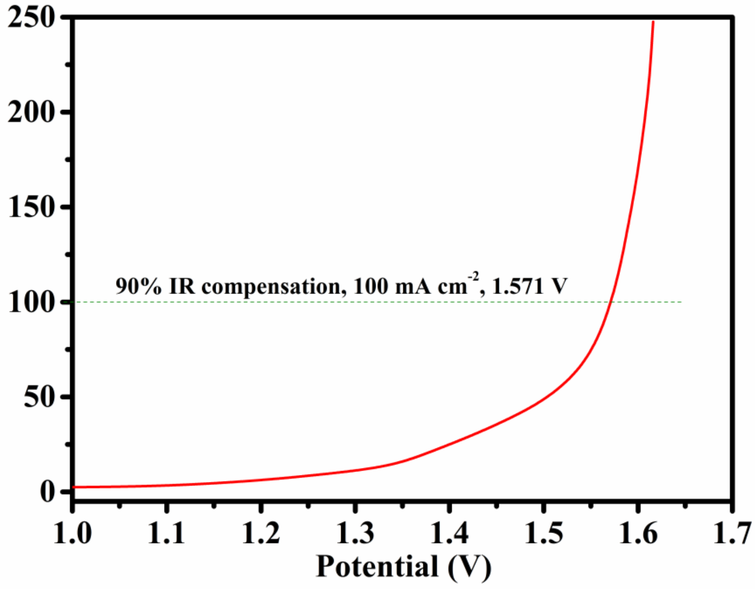

Fig. S35 LSV curve of 5#N with 90% IR compensation.

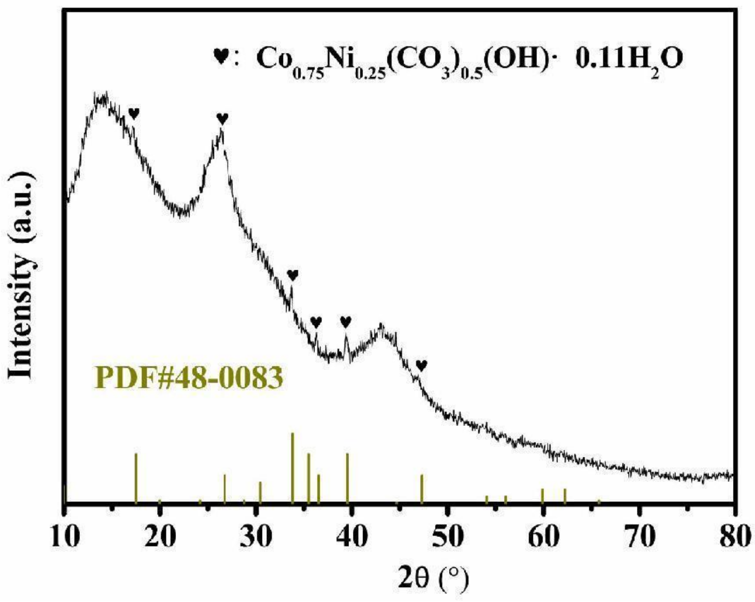

Fig. S36 XRD curve of tested 5#N.

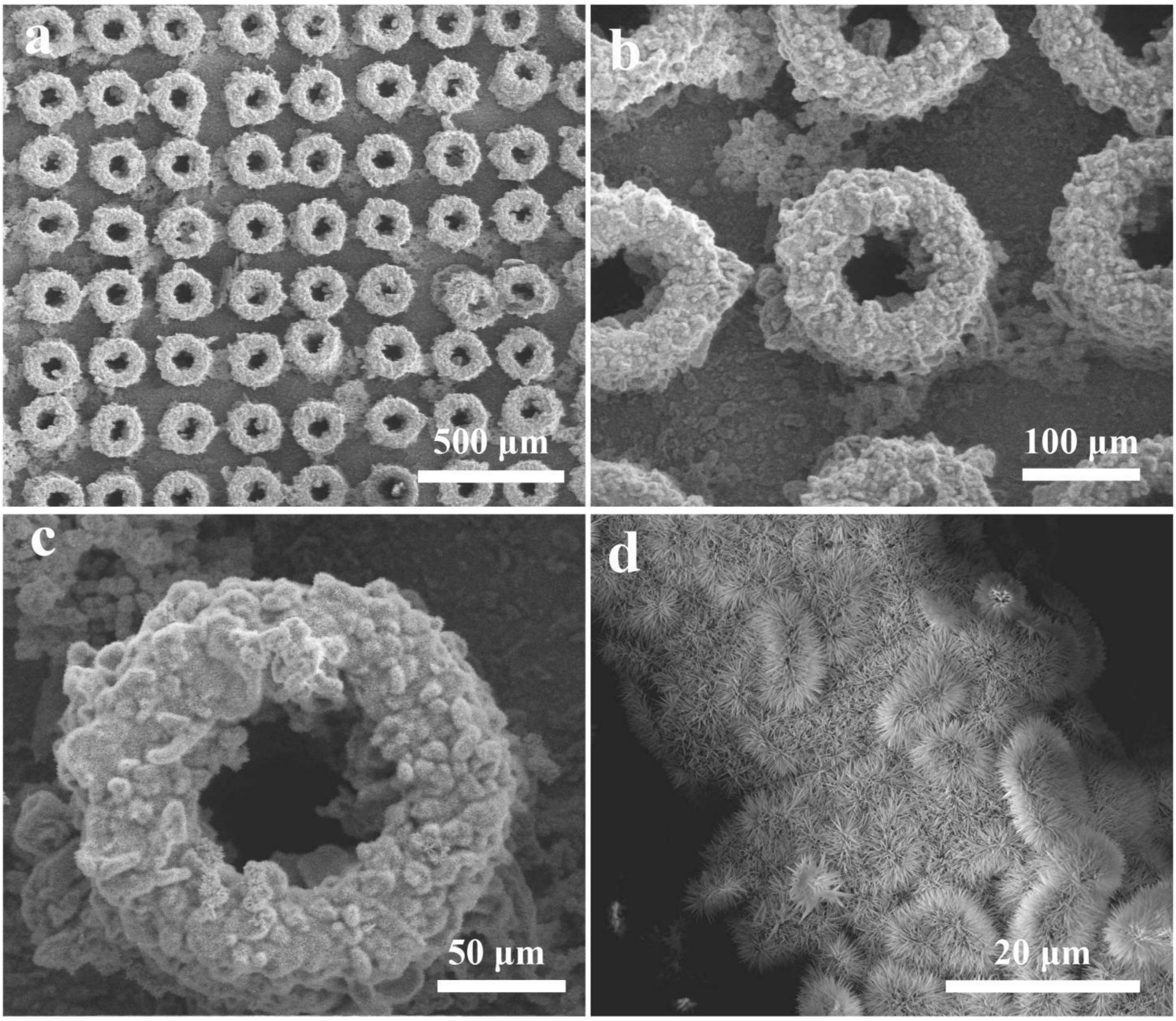

Fig. S37 SEM images of tested 5#N with different magnifications.

References

1.A. Liao, Y. Zhou, L. Xiao, C. Zhang, C. Wu, A.M. Asiri, M. Xiao, Z. Zou, Nanoscale 2018, 11, 109-114.

2. Y.Y. Zhou, N.X. Jin, Y.B. Ma, Y.S. Cui, L.N. Wang, Y.W. Kwon, W.K. Lee, W. Zhang, H.X. Ge, J. Zhang, Adv. Mater. 2022, 2209500.

3.S.L. Wang, G.L. Lu, W.H. Tang, Powder Diffraction 2010, 25, S7-S10.

4.O.L. Bacq, A. Pasturel, Philos. Mag. 2005, 85, 1747-1754.

5.P. Novák, F.R. Wagner, J. Magn. Magn. Mater. 2004, 272-276, E269-E270.

5. Y. Cai, Y. Li, P. Nordlander, P.S. Cremer, Nano Lett. 2012, 12, 4881-4888.

6. Y.Z. Tsai, Y.T. Liu, Y.L. Wang, L.C. Chang, S.Y. Hsu, Geofluids 2018, 2018, 1-11.

7. S. Gorsse, B. Ouvrard, M. Gouné, A. Poulon-Quintin, J. Alloys Compd. 2015, 633, 42-47.

8. A. Lund, Y. Wu, B. Fenech-Salerno, F. Torrisi, T.B. Carmichael, C. Muller, MRS Bull. 2021, 46, 491-501.

9. M.D. Demetriou, M.E. Launey, G. Garrett, J.P. Schramm, D.C. Hofmann, W.L. Johnson, R.O. Ritchie, Nat. Mater. 2011, 10, 123-128.

10. P. Kumar, S. Penta, S.P. Mahapatra, Integr. Ferroelectr. 2019, 202, 41-51.

11. A.M. Diez-Pascual, C. Sainz-Urruela, C. Valles, S. Vera-Lopez, M.P.S. Andres, Nanomaterials-Basel 2020, 10, 239.

12. K. Piela, K. Hołderna-Natkaniec, M. Baranowski, T. Misiaszek, J. Baran, M. Magdalena Szostak, J. Mole. Struc. 2013, 1033, 91-97.

13. S.A. Markarian, Z.K. Papanyan, G.A. Shahinyan, J. Solution Chem. 2020, 49, 1094-1106.

14. G. Mandal, S. Bhattacharya, J. Chowdhury, T. Ganguly, J. Mole. Struc. 2010, 964, 9-17.

15. L. Zeng, M. Xie, Q. Zhang, Y. Kang, X. Guo, H. Xiao, Y. Peng, J. Luo, Carbohydr. Polym. 2015, 123, 89-98.

16. J. Li, L. Wang, L. Dai, L. Zhong, B. Liu, J. Ren, Y. Xu, J. Mater. Sci. 2017, 53, 1874-1886.

17. X. Cao, Y. Liu, Y. Zhong, L. Cui, A. Zhang, J.M. Razal, W. Yang, J. Liu, J. Mater. Chem. A 2020, 8, 1837-1848.

18. A. Karmakar, S.K. Srivastava, ACS Appl. Energy Mater. 2020, 3, 7335-7344.

19. H. Gu, Q. Zhong, Y. Zeng, S. Zhang, Y. Bu, J. Colloid Interface Sci. 2020, 573, 299-306.

20. Y. Miao, T. Wang, J. Hua, K. Liu, Z. Hu, Q. Li, M. Zhang, Y. Zhang, S. Liu, X. Xue, J. Qi, F. Wei, Q. Meng, Y. Ren, B. Xiao, Y. Sui, P. Cao, ACS Appl. Mater. Interfaces 2021, 13, 39205-39214.

21. A. Karmakar, S.K. Srivastava, Int. J. Hydrogen Energy 2022, 47, 22430-22441.

22. M.C. Hsiao, S.H. Liao, M.Y. Yen, P.I. Liu, N.W. Pu, C.A. Wang, C.C. Ma, ACS Appl. Mater. Interfaces 2010, 2, 3092-3099.

23. W.M. Silva, H. Ribeiro, L.M. Seara, H.D.R. Calado, A.S. Ferlauto, R.M. Paniago, C.F. Leite, G.G. Silva, J. Braz. Chem. Soc. 2012, 23, 1078-1086.

24. K.H. Kim, Y.H. Choi, Mater. Res. Express 2022, 9, 034001.

25. Y. Wang, Z. Zhong, Y. Chen, C.T. Ng, J. Lin, Nano Res. 2011, 4, 695-704.

26. D.J. Morgan, C-J. Carbon Res. 2021, 7, 51.

27. D.V. Sivkov, O.V. Petrova, S.V. Nekipelov, A.S. Vinogradov, R.N. Skandakov, K.A. Bakina, S.I. Isaenko, A.M. Ob'edkov, B.S. Kaverin, I.V. Vilkov, V.N. Sivkov, Appl. Sci. 2022, 12, 7744.

28. J.K. Norsko, Rep. Prog. Phys. 1999, 53, 1253-1295.

29. R.A. Mάrquez, K. Kawashima, Y.J. Son, R. Rose, L.A. Smith, N. Miller, O.A. C. Jaim, H. Celio, C. B. Mullins, ACS Appl. Mater. Interfaces 2022, 14, 42153-42170.

30. R.A. Marquez-Montes, K. Kawashima, Y.J. Son, J.A. Weeks, H.H. Sun, H. Celio,V.H. Ramos-S´ anchez, C.B. Mullins, J. Mater. Chem. A, 2021, 9, 7736-7749.

31. J.K.Nørskov. Prog. Surf. Sci. 1991, 38, 103-144.

32. N.S. Gultom, C.H. Li, D.H. Kuo, H. Abdullah, ACS Appl. Mater. Interfaces 2022, 14, 39917-39926.

33. H. Zhang, Y. Dong, S. Zhao, G. Wang, P. Jiang, J. Zhong, Y. Zhu, Appl. Catal. B: Environ. 2020, 261, 118233.
